# Exploring the Role of Macrophage Marker CD68 in Pediatric Acute Myeloid Leukemia

**DOI:** 10.3390/ijms27115136

**Published:** 2026-06-05

**Authors:** Laurens Van Camp, Jolien Vanhooren, Barbara Depreter, Mattias Hofmans, Inge D’Hont, Christophe Chantrain, Laurence Dedeken, An Van Damme, Anne Uyttebroeck, Tim Lammens, Barbara De Moerloose

**Affiliations:** 1 Department of Pediatric Hematology-Oncology and Stem Cell Transplantation, Ghent University Hospital, 9000 Ghent, Belgium; laurens.vancamp@ugent.be (L.V.C.);; 2 Department of Internal Medicine and Pediatrics, Ghent University, 9000 Ghent, Belgium; 3 Cancer Research Institute Ghent (CRIG), 9000 Ghent, Belgium; 4 Department of Laboratory Medicine, AZ Delta General Hospital, 8800 Roeselare, Belgium; 5 Department Pharmaceutical Sciences (FARM), University Hospital Brussels, 1090 Brussels, Belgium; 6 Department of Diagnostic Sciences, Ghent University, 9000 Ghent, Belgium; 7 Department of Laboratory Medicine, Ghent University Hospital, 9000 Ghent, Belgium; 8 Division of Pediatric Hematology-Oncology, Centre Hospitalier Chrétien (CHC), MontLégia, 4000 Liege, Belgium; 9 Department of Pediatric Hematology-Oncology, Hôpital Universitaire des Enfants Reine Fabiola, 1020 Brussels, Belgium; 10 Department of Pediatric Hematology Oncology, Cliniques Universitaires Saint-Luc, 1200 Brussels, Belgium; 11 Department of Pediatrics, University Hospital Gasthuisberg, 3000 Leuven, Belgium

**Keywords:** pediatric acute myeloid leukemia, AML, CD68, macrophage marker, targeted therapy, biology

## Abstract

Pediatric acute myeloid leukemia (pedAML) is a childhood malignancy with relapse rates of approximately 30%. CD68, a macrophage marker involved in phagocytosis and macrophage recruitment, may contribute to AML biology. We analyzed *CD68* expression using the TARGET database and performed survival analyses, mRNA/protein profiling, and functional assays in AML cell lines, pedAML samples, and cord blood samples. High *CD68* transcript levels correlated with *KMT2A*-rearrangements and inversion 16. Survival analysis showed that high *CD68* predicted worse event-free survival, though not independently in a multivariate analysis. Flow cytometry confirmed higher CD68 expression in 7/8 pedAML samples compared to cord blood samples. Functionally, *CD68* knockdown reduced proliferation and increased drug sensitivity, while overexpression promoted growth and resistance. Gene set enrichment analysis (GSEA) indicated enrichment of MAPK signaling, AP-1–mediated stress response, and epithelial–mesenchymal transition (EMT)/migration-associated pathways in *CD68*-high models. Together, these findings suggest that CD68 contributes to a pro-tumorigenic and stress-adaptive phenotype in pedAML and may represent a biologically relevant therapeutic target.

## 1. Introduction

Pediatric acute myeloid leukemia (pedAML) is a heterogeneous hematological malignancy in childhood whose survival rate has considerably improved in recent decades [1,2,3]. However, approximately 30% of patients with pedAML still experience relapse, mostly attributed to resistant residual leukemic stem cells (LSC) [4,5]. In addition, the current therapy approaches are associated with severe life-long toxicities seriously impacting the quality of life of survivors [6,7]. Targeting the LSC is one way to overcome the high relapse rate; decades of molecular studies have not yielded a universal LSC-specific marker, given the heterogeneity of the disease [8,9].

At the onset of AML, the dynamics of the bone marrow (BM) niche change as the LSCs outgrow the hematopoietic stem cells (HSCs). Cytokine signaling and growth factors are taken over, the vascular permeability is increased, and the interaction with neighboring cells is altered. LSCs are able to shift this supportive microenvironment from HSC-supportive to LSC-supportive, leading to leukemic growth and disrupted hematopoiesis [10,11,12]. Therapeutics targeting the altered microenvironment have been shown to exhibit a synergistic effect in other cancers when combined with conventional therapy [13,14]. One of the major players in the microenvironment is tumor-associated macrophages (TAM), and although believed to only play a role in solid tumors, recent studies have demonstrated the leukemia-preserving and -protecting role of TAMs, or leukemia-associated macrophages (LAM), in hematological diseases as well [15,16].

CD68 (LAMP4), a pan-macrophage marker expressed in both M1 and M2 macrophages [17], belongs to a growing family of hematopoietic mucin-like molecules known as lysosomal/endosomal-associated membrane glycoproteins (LAMPs) [18]. It binds to lectins through a heavily glycosylated extracellular domain and, as a member of the scavenger receptor family, also plays a role in clearing cell debris, regulating phagocytosis, and recruiting macrophages [17,18]. Although well-known for its high levels of expression in human monocytes and tissue macrophages [17], higher expression was found in CD34+ AML blast cells compared to normal CD34+ BM cells. In addition, only a subset of CD19+ B cells weakly express CD68, with no expression in CD3+ T cells or CD56+ NK cells [19]. Interestingly, CD68 is the most commonly expressed marker in myeloid sarcoma and was suggested as a universal marker to monitor minimal residual disease (MRD) in pediatric and adult AML [20,21]. Higher transcript levels of *CD68* were also observed in the LSC compartment of diagnostic patients with pedAML when compared with HSC [5]. Here, we investigated the role of CD68 in (ped)AML and explored its potential as a therapeutic target. 

## 2. Results

### 2.1. CD68 Expression and Clinical Characteristics

**Figure 1:**
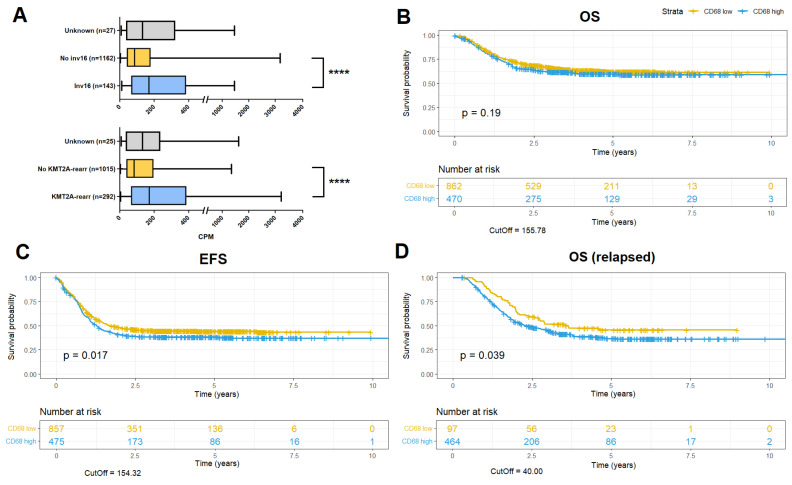
(**A**) Counts per Million (CPM) values of *CD68* in patients with pedAML from the TARGET database (*n* = 1332), categorized by cytogenetic abnormalities (**** = *p* < 0.0001). (**B**) Kaplan–Meier survival analysis showing the overall survival (OS) in years of patients with pedAML from the TARGET database (*n* = 1332), based on high (blue) and low (yellow) *CD68* expression (*p* = 0.19, cut-off CPM =155.78). (**C**) Kaplan–Meier survival analysis showing the event-free survival (EFS) in years for patients with pedAML from the TARGET database (*n* = 1332), based on high (blue) and low (yellow) *CD68* expression (*p* = 0.017; cut-off CPM = 154.32). (**D**) Kaplan–Meier survival analysis showing the overall survival (OS) in years of all relapsed patients with pedAML from the TARGET database (*n* = 561), based on high (blue) and low (yellow) *CD68* expression (*p* = 0.039; cut-off CPM = 40.0).

As CD68 was previously observed in at least a subset of adult patients with AML and also described in pedAML, we sought to identify the clinical and molecular features associated with high *CD68* expression in pedAML. To this end, 1332 patients with pedAML from the TARGET AML study were analyzed, including full RNA sequencing and clinical characteristics (Supplementary Materials Tables S1 and S2). According to cytogenetic or molecular aberrations, significantly higher *CD68* expression could be observed in patients with an inv(16), patients carrying a *KMT2A* rearrangement, patients with *WT1* overexpression, and those carrying a *c-KIT* mutation (Figure 1A, Supplementary Materials Figure S1). A higher median expression of *CD68* in patients with inv(16) or *KMT2A*-rearrangement could be confirmed in the published pediatric AML dataset by den Boer and colleagues (*n* = 237, Balgobind/den Boer et al.) (Supplementary Materials, Figure S2A) [22]. However, no significantly higher *CD68* expression in patients carrying a *c-Kit* mutation could be confirmed in the same dataset (Supplementary Materials, Figure S2B).

Furthermore, no significant differences in median *CD68* expression were observed according to sex, blast percentage in the BM at diagnosis, or relapse incidence (*p* = 0.1741, *p* = 0.3519, and *p* = 0.1892, respectively). According to age, the median *CD68* expression was higher in the age category of <2 years old compared to 2–10 year old patients (*p* = 0.01) but lower compared to patients of 10–16 years old and >16 years old (*p* = 0.0032 and *p* = 0.0139, respectively). A significantly higher median *CD68* expression was seen in patients with a WBC count of >100,000/µL at diagnosis compared to a WBC count of 10,000–100,000/µL and <10,000/µL (*p* = 0.0047 and *p* < 0.0001, respectively). Patients with 50–75% blast count in the peripheral blood at diagnosis had a higher median *CD68* expression (*p* = 0.0009, *p* = 0.2497, and *p* = 0.0022 in >75%, 25–50%, and <25% blast counts, respectively). A significantly lower median *CD68* expression was seen in patients with CNS invasion (*p* < 0.0001) (Supplementary Materials, Figure S3A–D).

Finally, Kaplan–Meier survival analysis showed no significant difference in OS between patients based on high or low *CD68* expression (*p* = 0.19, Figure 1B), while the EFS was significantly lower in patients with higher *CD68* expression compared to lower *CD68* expression (*p* = 0.017, Figure 1C). Remarkably, the OS of all relapsed patients (*n* = 561) in the TARGET dataset was also significantly lower in *CD68*-high compared to *CD68*-low expressing patients at diagnosis (*p* = 0.039, Figure 1D).

**Figure 2:**
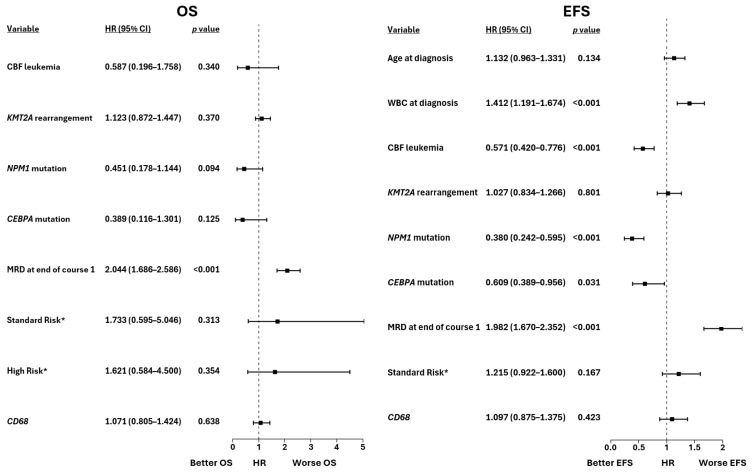
Independent prognostic analysis of *CD68*. Forest plots of multivariate independent Cox regression analyses of the overall survival (OS) and event-free survival (EFS), including *CD68* expression and other characteristics. The dotted line represents the reference (HR = 1.0). * according to the COG protocols. Abbreviations: HR, hazard ratio; CI, confidence interval; WBC, white blood cell; CBF, core-binding factor; MRD, minimal residual disease.

Additionally, to explore the independent prognostic potential of *CD68* expression in this pedAML TARGET cohort, a univariate Cox regression analysis was performed, showing no significant differences in OS, nor EFS (Supplementary Materials, Figure S4). Consequently, we performed a multivariate Cox regression analysis, which confirmed that *CD68* is not an independent marker for OS, nor EFS (*p* = 0.638, HR 1.071 (0.805–1.424) and *p* = 0.423, HR 1.097 (0.875–1.375), respectively; Figure 2).

**Figure 3:**
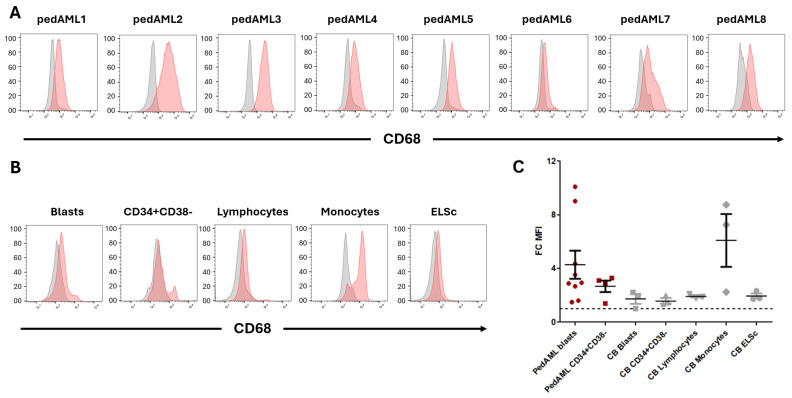
(**A**) Intracellular staining of CD68 on flow cytometry of pedAML samples (*n* = 8) in red, with the isotype control in gray. Counts are displayed as a percentage compared to the maximal count. (**B**) Intracellular staining of CD68 on flow cytometry of different fractions (*n* = 5) of a cord blood (CB) sample in red, with the isotype control in gray. Counts are displayed as a percentage compared to the maximal count. (**C**) Fold Change (FC) of the Median Fluorescence Intensity (MFI) of intracellular staining of CD68 in pedAML leukemic blast fractions (*n* = 8) in red, and cord blood fractions (blasts, CD34+CD38− cells, lymphocytes, monocytes, and embryonic-like stem cells) in gray, compared to the isotype control. The dotted line represents FC MFI = 1, meaning no expression compared to the isotype.

Next, we investigated the protein expression of CD68 using flow cytometry in bulk BM mononuclear cells of pedAML samples (*n* = 8), using the same workflow as previously described [23]. The patient characteristics and the leukemia-associated immunophenotype (LAIP) at diagnosis can be found in the same article [23]. Briefly, leukemic samples were gated on the LAIP at diagnosis before evaluating the CD68 expression (Figure 3A & Supplementary Materials Table S3 and Supplementary Methods Table S6). Three CB samples served as healthy controls for the blast population (Figure 3B). CB samples were gated on CD45 expression and SSC-A to differentiate the blast population from monocytes, lymphocytes, and embryonic-like stem cells (ELSc). Average of the median fluorescence intensity (MFI) of CD68 expression in the leukemic blasts was significantly higher compared to the CB blast and CB HSC CD34+CD38− fractions (Figure 3C, *p* = 0.049 and *p* = 0.024, respectively), while expression in the LSC CD34+CD38− fractions was also higher compared to CB blast and CD34+CD38− fractions but not significantly (both *p* = 0.23). Only in CB monocytes, the median MFI of CD68 expression was higher than in leukemic blasts or leukemic CD34+CD38− fractions, but not significantly (Figure 3C, *p* = 0.92 and *p* = 0.40, respectively).

To distinguish between normal and myeloid cells, we analyzed *CD68* expression in the single-cell RNA sequencing dataset of van Galen et al. [24]. Higher *CD68* expression was found in all malignant fractions compared to their normal counterparts (Supplementary Materials, Figure S5). However, the *CD68* expression in normal myeloid cells was higher compared to malignant hematopoietic stem cell and progenitor-like (HSC/Prog) cells and to malignant granulocyte-macrophage progenitor (GMP) cells, predicting toxicity towards normal myeloid cells when targeting CD68.

### 2.2. The Functional Role of CD68

To elucidate the role of CD68 in AML, we first assessed expression levels in 21 hematological cell lines using RT-qPCR, Western blot, and flow cytometry. CD68 expression on both RNA and protein levels was significantly higher in AML cell lines compared to other hematological cell lines (Supplementary Materials, Figure S6A–C). RNA expression levels were in agreement with the data obtained through DepMap (Spearman r = 0.744; Supplementary Figure S7). Based on both transcript and protein expression level, we selected THP-1 and MOLM-13 to generate KD models, whereas HL-60 and KASUMI-1 were used to generate OE models. Knockdown and overexpression were confirmed on both mRNA and protein levels (Supplementary Materials, Figures S8A–H and S9A–H).

**Figure 4:**
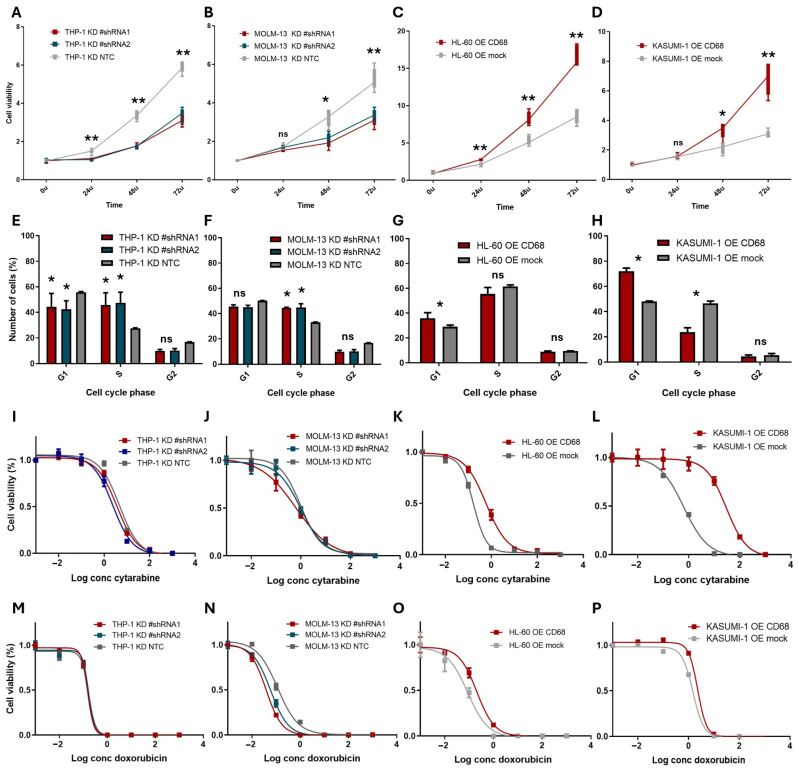
Proliferation assay (**A**–**D**), cell cycle assay (**E**–**H**), cytotoxicity to cytarabine (**I**–**L**) and to doxorubicin (**M**–**P**) of 2 THP-1*^CD68^* ^KD^, 2 MOLM-13*^CD68^* ^KD^, and HL-60*^CD68^* ^OE^ and KASUMI-1*^CD68^* ^OE^ vs. THP-1^NTC^, MOLM-13^NTC^, and HL-60^mock^ and KASUMI-1^mock^, respectively. Experiments were performed twice independently, with six technical replicates for proliferation/cytotoxicity assays and three technical replicates for cell cycle assays (* = *p* < 0.05, ** = *p* < 0.01, ns = non-significant).

Interestingly, *CD68* KD negatively impacted the proliferation of THP-1 and MOLM-13 cells and blocked cells in the S-phase (*p* < 0.0001 and *p* = 0.024 for THP-1 and *p* < 0.001 and *p* = 0.114 for MOLM-13, respectively, Figure 4A,B,E,F). Next, sensitivity to cytarabine upon KD of *CD68* in the THP-1 and MOLM-13 cell lines was not significantly different (logIC50 THP-1*^CD68^* ^KD1^ = 0.583 vs. THP-1^NTC^ = 0.696, *p* = 0.201; logIC50 MOLM-13*^CD68^* ^KD1^ = −1.470 vs. MOLM-13^NTC^ = −0.938, *p* = 0.195; Figure 4I,J). However, while the sensitivity to doxorubicin was also not statistically different between the THP-1 KD cell lines and the THP-1^mock^ cell line (logIC50 THP-1*^CD68^* ^KD1^ = −0.632 vs. THP-1^NTC^ = −0.675, *p* = 0.645; Figure 4M), MOLM-13*^CD68^* ^KD1^ cells were significantly more sensitive to doxorubicin (logIC50 MOLM-13*^CD68^* ^KD1^ = −1.470 vs. MOLM-13^NTC^ = −0.987, *p* < 0.001; Figure 4N). In contrast, overexpression of *CD68* increased proliferation of HL-60 and KASUMI-1 cells (both *p* < 0.001), blocked them in the G1 phase (*p* = 0.186 and *p* < 0.001, respectively) and increased their resistance to cytarabine (logIC50 HL-60*^CD68^* ^OE^ = −0.229 vs. HL-60^mock^ = −0.808, *p* < 0.0001 and logIC50 KASUMI-1*^CD68^* ^OE^ = 0.466 vs. KASUMI-1^mock^ = −1.18, *p* < 0.001; Figure 4C,D,G,H,K,L). Also, OE of *CD68* in HL-60 and KASUMI-1 rendered the cells more resistant to doxorubicin (logIC50 HL-60*^CD68^* ^OE^ = −0.685 vs. HL-60^mock^ = −1.070, *p* = 0.018; logIC50 KASUMI-1*^CD68^* ^OE^ = 0.457 vs. KASUMI-1^mock^ = −0.231, *p* = 0.003; Figure 4O,P). Furthermore, at 48 h, no significant difference in proportion of live cells and proportion of early and late apoptotic cells was observed upon knockdown (*p* = 0.84 and *p* = 0.38, for THP-1 and MOLM-13, respectively, Supplementary Materials, Figure S10A,B). Similarly, OE models also showed no significant difference (*p* = 0.84 and *p* = 0.07 for HL60 and KASUMI-1, respectively; Supplementary Materials, Figure S10C,D). Together, these data thus confirm that *CD68* KD cells tend to proliferate more slowly instead of dying more, while *CD68* OE cells proliferate faster instead of dying less.

### 2.3. Pathway Analysis of CD68 KD and OE

**Figure 5:**
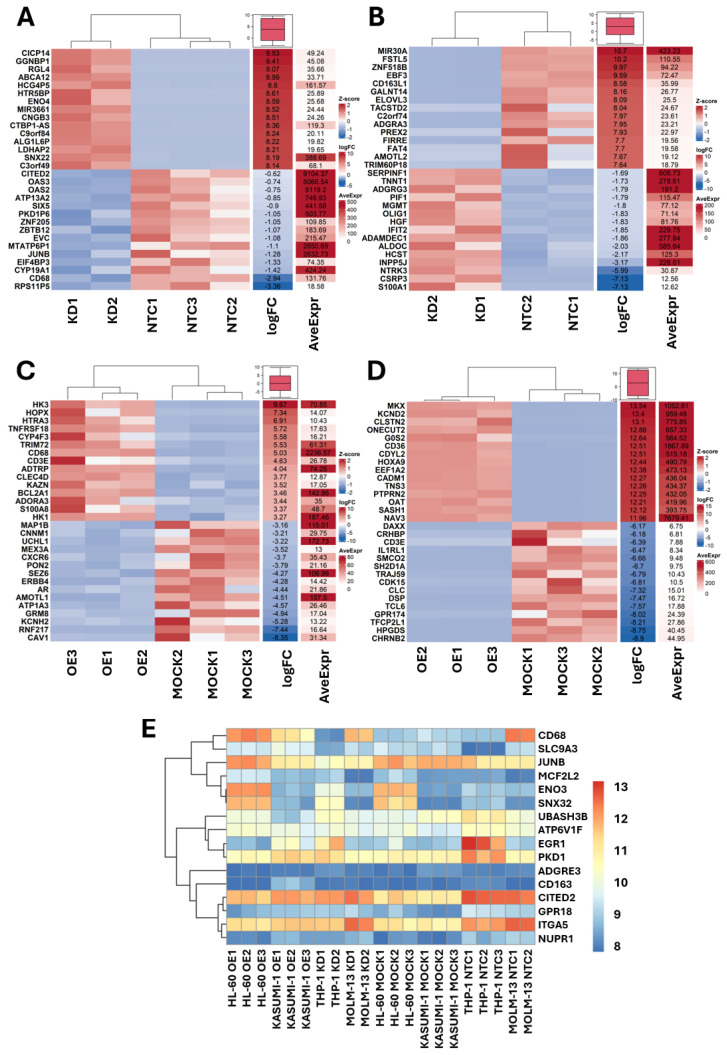
Heatmap of the top 15 significantly upregulated and downregulated genes of (**A**) THP-1*^CD68^* ^KD1^ compared to THP-1^NTC^, (**B**) MOLM-13*^CD68^* ^KD1^ compared to MOLM-13^NTC^, (**C**) HL-60*^CD68^* ^OE^ compared to the HL-60^mock^, and (**D**) KASUMI-1*^CD68^* ^OE^ compared to the KASUMI-1^mock^. (**E**) Heatmap of the core overlap genes (*n* = 16) between the 4 models, variance-stabilized and scaled across all samples.

RNA sequencing and differential expression analysis between *CD68* shRNA1-THP1 (THP-1*^CD68^* ^KD1^) and THP-1^NTC^ revealed 1568 genes being significantly upregulated and 47 genes significantly downregulated (adjusted *p*-value < 0.05), among which a significant KD of *CD68* (adjusted *p*-value = 3.05 × 10^−11^, log FC −2.96; Figure 5A, Supplementary Materials, Figure S11A). Similarly, RNA sequencing and differential expression analysis between *CD68* shRNA1-MOLM13 (MOLM-13*^CD68^* ^KD1^) and MOLM-13^NTC^ revealed 62 genes being significantly upregulated and 53 genes significantly downregulated (adjusted *p*-value < 0.05), among which a significant KD of *CD68* (adjusted *p*-value = 0.00055, log FC −0.76; Figure 5B, Supplementary Materials, Figure S11B). Next, the differentially expressed genes of HL-60*^CD68^* ^OE^ compared to HL-60^mock^ and KASUMI-1*^CD68^* ^OE^ compared to KASUMI-1^mock^ were identified and plotted in a heatmap and a volcano plot (Figure 5C,D, Supplementary Materials, Figure S11C,D). A significant overexpression of *CD68* was observed in HL-60 (adjusted *p*-value = 2.1 × 10^−208^, log FC 5.03) as well as a significant upregulation and downregulation of 476 and 358 genes, respectively. In KASUMI-1, a significant overexpression of *CD68* was observed (adjusted *p*-value = 1.40 × 10^−17^, log FC 2.95) as well as a significant upregulation and downregulation of 2894 and 2155 genes, respectively. Principal component analysis plots of the KD and OE models are shown in the Supplementary Materials (Figure S12).

GSEA of the *CD68* overexpression models revealed enrichment in pathways related to MAPK signaling, TGF-β signaling, and cell adhesion, alongside transcriptional and metabolic programs, including mTORC1 signaling and hypoxia-related pathways. In contrast, pathways associated with oxidative phosphorylation, cell cycle progression, and MYC targets were significantly downregulated, suggesting a shift towards a less oxidative and more stress-adapted cellular state (Supplementary Materials Table S4). In *CD68* knockdown models, enrichment was observed for pathways related to oxidative phosphorylation, TP53 signaling, and antigen processing and presentation, indicative of increased mitochondrial activity and cellular stress responses (Supplementary Materials Table S5). Notably, several pathways showed opposite regulation between overexpression and knockdown models, particularly oxidative phosphorylation and stress-related signaling, supporting a role for CD68 in regulating metabolic and stress-adaptive transcriptional programs. An overview figure of the different enriched pathways across all models is shown in Supplementary Materials Figure S13.

## 3. Discussion

As pedAML is a heterogeneous hematological childhood malignancy with a high relapse rate, more targeted therapy approaches are needed. Significantly higher expression of *CD68* was seen in patients with pedAML carrying inv(16) or KMT2A rearrangements, which was in line with data on the protein level by Coustan-Smith et al. [20]. In a univariate analysis, *CD68* expression was shown to impact EFS. However, *CD68* expression was not independently prognostic for OS or EFS in patients of the TARGET pedAML cohort, nor was the presence of *KMT2A* rearrangements [25,26]. Although Liu et al. showed that adult patients with high expression of *CD68* show a low rate of complete remission, suggesting that the expression level of *CD68* is correlated with treatment response, we could not confirm this correlation in a pedAML cohort [27]. This discrepancy may partly reflect biological differences between pediatric and adult AML, which are characterized by distinct mutational landscapes, differentiation states, and microenvironmental dependencies [28]. Whereas adult AML more frequently harbors age-related clonal hematopoiesis-associated mutations and inflammatory signaling signatures, pediatric AML is often driven by fusion oncogenes, such as *KMT2A* rearrangements, that may influence the functional role and prognostic impact of *CD68* differently [28]. However, despite the absence of a significant association with treatment response in our cohort, our functional data demonstrating increased proliferation and decreased drug sensitivity upon *CD68* overexpression still support a biologically relevant role for CD68 in leukemic cell fitness. Together, these findings suggest that the prognostic implications of *CD68* expression may differ between adult and pedAML contexts, while its contribution to leukemic biology may remain conserved. In addition, although the TARGET dataset lacks data on *RAS* mutations, the dataset of den Boer et al. showed significantly higher *CD68* expression in patients with *NRAS* or *KRAS* mutations, known to be co-occurring with KMT2A rearrangements [22,25,29,30,31,32]. As the role of the AP-1 family of transcription factors was recently uncovered in *KMT2A*-rearranged AML with *NRAS* mutations, *CD68* might be involved in this transcriptional network, supported by the differential expression of AP-1-related genes such as *EGR1*, *NUPR1*, *ITGA5*, and *JUNB* in our generated overexpression and knockdown datasets [33]. Importantly, we also observed elevated *CD68* expression in additional high-risk molecular subgroups, including *KMT2A*-*AFDN* and *NUP98*-rearranged AML, both of which are associated with poor clinical outcomes [29]. These subtypes are characterized by transcriptional reprogramming involving *HOXA*-driven gene expression, enhanced proliferative signaling, and increased cellular plasticity. The overlap between these known oncogenic programs and the pathways identified in our *CD68*-associated transcriptomic analyses, including MAPK signaling, cell cycle regulation, and stress response pathways, suggests that *CD68* expression may mark or contribute to a broader aggressive leukemic cell state rather than being restricted to a single cytogenetic subgroup. Moreover, the lower *CD68* expression observed in *KMT2A*-*MLLT11* leukemias suggests that not all *KMT2A*-rearranged AMLs share a common differentiation or inflammatory phenotype, highlighting biological heterogeneity within this subgroup. Nevertheless, the precise role of *CD68* in the interplay of *KMT2A* or *NUP98* rearrangements and RAS signaling remains to be further elucidated. Interestingly, while *CD68* expression is elevated in core binding factor AML, including inv(16) cases, this does not necessarily confer an adverse prognosis. However, in the presence of co-occurring mutations such as *KIT* or *RAS*, which activate MAPK and AP-1 signaling, *CD68* expression may instead reflect a more proliferative and therapy-resistant leukemic state.

Compared with normal counterparts of CB samples, CD68 expression in patients with pedAML was higher on the protein level, which is in agreement with the transcript single cell data of van Galen et al. and previous data of Coustan-Smith et al. [20,24]. Our findings are also in line with the early observations by Strobl et al., who demonstrated broad CD68 expression across normal and malignant myeloid differentiation and reported increased CD68 levels in AML blasts compared with normal CD34+ hematopoietic progenitors [19]. In that study, CD68 was primarily interpreted as a differentiation-associated myeloid marker, reflecting early granulo-monocytic commitment rather than a functional oncogenic driver. Although the expression is known to be mostly intracellular in lysosomes and endosomes, surface expression has been described in cells with high endogenous CD68 expression, which may allow CD68 to influence extracellular interactions, including adhesion and migration [34]. Indeed, our transcriptomic analyses revealed enrichment of pathways related to cell adhesion and extracellular matrix interaction, including genes such as *ITGA5* and *SPON2*, suggesting a potential role in leukemic cell trafficking. Moreover, we found enrichment of epithelial-to-mesenchymal transition (EMT)-like gene signatures, which, although classically described in solid tumors, have been associated with increased migratory and invasive capacity in hematological malignancies [35,36]. Such a phenotype may contribute to the development of extramedullary disease manifestations, including myeloid sarcomas, in which *CD68* expression is typically elevated, and the MAPK/ERK pathway is frequently activated [25,36]. Nevertheless, targeting CD68 predicts toxicity towards normal myeloid cells, especially monocytes and macrophages. While monocytes are short-lived and undergo spontaneous apoptosis on a daily basis, macrophages have a longer lifespan, ranging from months to years [37,38,39]. In the case of toxicity towards monocytes and macrophages, the former will be regenerated out of HSCs that are spared by more targeted HSC-saving therapies, and the latter is expected to be replenished after some time, thereby avoiding long-term immunosuppression [40].

Across models, a core set of genes was identified displaying bidirectional regulation, including *CD163*, *CITED2*, *EGR1*, *ITGA5*, and *NUPR1*, which are associated with immediate-early immune response, MAPK signaling, interferon pathways, and cellular stress adaptation [15,40,41,42,43,44,45]. Gene set enrichment analysis further supported a role for *CD68* in key oncogenic pathways, including MAPK signaling, cell cycle regulation, and metabolic processes, whereas knockdown of *CD68* resulted in enrichment of pathways associated with cellular stress responses and apoptosis. Together, these findings suggest that *CD68* may act upstream or in parallel to major signaling pathways that regulate leukemic cell survival and proliferation [46]. Our functional assays of *CD68* KD and OE further support this hypothesis. *CD68* knockdown cells showed reduced proliferation and impaired cell cycle progression with S phase accumulation, whereas *CD68* overexpression resulted in enhanced proliferative capacity and reduced sensitivity to cytarabine and doxorubicin, which is in line with our transcriptomic findings. These findings, therefore, extend the descriptive observations of Strobl et al. by supporting a functional contribution of *CD68* to AML cell fitness, suggesting that *CD68* expression may be linked to broader transcriptional programs involved in leukemic adaptation and immune-interactive behavior, rather than solely reflecting myeloid maturation status [19].

Beyond its intrinsic effects on leukemia cells, CD68 may also contribute to the establishment of an immune-evasive leukemic microenvironment. AML cells actively remodel the immune niche through the secretion of immunosuppressive cytokines, induction of T-cell dysfunction, and modulation of myeloid cell populations, ultimately impairing anti-leukemic immunity [10]. Although CD68 is classically considered a macrophage-associated lysosomal glycoprotein, increased CD68 expression on AML blasts may reflect the acquisition of macrophage-like immune-regulatory properties that facilitate immune escape. In solid tumors, CD68-positive macrophage infiltration has consistently been associated with T-cell suppression, tumor progression, and poor clinical outcome, supporting a broader role for *CD68*-associated signaling in shaping suppressive microenvironments [13,15,47]. Furthermore, activation of pathways identified in our GSEA analysis, including MAPK/AP-1 signaling and EMT/migration-associated programs, has previously been linked to inflammatory cytokine production and immune suppression in AML [48,49]. Future studies should therefore investigate whether *CD68* knockdown in AML cells reduces suppression of T-cell proliferation or effector function in PBMC co-culture systems compared with control conditions. Such experiments may provide further insight into whether CD68 contributes not only to leukemic cell fitness, but also to immune evasion mechanisms that could represent therapeutically targetable vulnerabilities.

This study had some limitations. First, flow cytometry validations were performed in a patient cohort that is not fully representative of the total patient population. Nonetheless, our results are in line with studies in different cohorts of patients with (ped)AML. Therefore, it would be of interest to validate these findings in a larger, prospective cohort. Second, although multiple cell line models were included to assess both *CD68* overexpression and knockdown, further validation in primary patient-derived samples or in vivo models would strengthen the conclusions. Moreover, since *CD68* knockdown and overexpression experiments were performed in different AML cell lines rather than within the same cellular model, direct comparison of *CD68*-dependent effects in an identical biological context was not feasible. As these cell lines differ in genetic background, molecular subtype, and differentiation state, part of the observed phenotypic variation may reflect intrinsic cell line–specific characteristics rather than exclusively *CD68*-mediated effects. Third, although cytarabine and doxorubicin are cornerstone drugs in AML treatment, it would be of great interest to perform a broader drug response profiling to assess the impact of *CD68* expression on sensitivity to multiple therapeutic agents, including inhibitors of the MAPK pathway, such as MEK inhibitors.

In conclusion, this study shows that macrophage marker *CD68* is highly expressed in pedAML compared to normal hematopoietic stem cells. Our combined transcriptomic and functional analyses suggest that *CD68* might contribute to leukemic cell survival, proliferation, and potentially migration through modulation of key oncogenic pathways. Targeting CD68, either directly or through its downstream pathways, may therefore represent a novel therapeutic strategy in both pediatric and adult AML.

## 4. Materials and Methods

### 4.1. Patient Samples

As previously described [23], stored bulk BM mononuclear cells from 8 patients with pedAML were selected based on cell availability (>10 × 10^6^ cells), approved by the Ethics Committee of the Ghent University Hospital (EC2015-1443 and EC2019-0294). Pediatric control samples and umbilical cord blood (CB) samples were collected to evaluate *CD68* expression in non-leukemic blood samples and predict potential toxicities to healthy blood cells. Fresh blood samples were collected from non-hematological, non-oncological, non-infectious pediatric patients. CB samples were obtained from the Red Cross Belgium after informed consent was given by the mother and served as a flow cytometry control for normal blasts. The Ethics Committee of the Ghent University Hospital approved the prospective collection and study (ONZ-2023-0314). All studies were conducted in accordance with the Declaration of Helsinki.

### 4.2. Analysis of Publicly Available Data

RNA sequencing data of BM and peripheral blood samples were obtained from patients enrolled in the Therapeutically Applicable Research to Generate Effective Treatments (TARGET) AML study (*n*  =  1332; https://portal.gdc.cancer.gov, accessed on 9 November 2023; study “TARGET-AML”). We included pedAML cases from COG studies CCG2961, AAML0531, and AAML1031.11–14. Raw counts were used to calculate the counts per million (CPM), normalizing for the sequencing depth across samples. Consequently, *CD68* expression was correlated with clinical characteristics at diagnosis, and the impact of *CD68* expression levels on survival was assessed. The transcripts exhibiting CPMs with a total sum across all patient samples < 1 were excluded from the analysis. Furthermore, the R2 Platform (https://r2.amc.nl, accessed on 19 December 2023) served to validate *CD68* expression in an independent pedAML cohort (Balgobind/den Boer et al.) [22]. Additionally, the dataset of van Galen et al. [24] was used to validate *CD68* expression in normal and malignant hematopoietic stem cells (HSCs), granulocyte-monocyte progenitor cells (GMPs), and myeloid cells. Finally, RNA sequencing data of a subset of hematological cell lines in the DepMap database were also analyzed (DepMap Portal, https://depmap.org/portal/interactive/, accessed on 19 December 2023).

### 4.3. Cell Lines, RNA Isolation, cDNA Synthesis, qPCR Analysis, Protein Extraction, Western Blot Analysis, and Flow Cytometry Analysis

All analyses and techniques were performed according to standard protocols [50], and a full description can be found in the Supplementary Information. Specifics on the used antibodies and the gating strategy for the flow cytometry experiments can be found in Supplementary Methods Table S6 and Supplementary Methods Figures S14–S16.

### 4.4. Generation of Overexpression (OE) and Knockdown (KD) Models

To overexpress *CD68*, a constitutive Lenti-hCMV-ORF-P2A-eGFP-IRES-Puromycin vector was used with an empty lentiviral open-reading frame vector as a control; both were provided as bacterial glycerol stocks (Transomic, Huntsville, AL, USA). For the knockdown models, three shERWOOD UltramiR short hairpin RNAs targeting *CD68*, and one non-targeting shRNA (NTC) were purchased as glycerol stocks at Transomic (TLHSU1452). The OE and KD vector backbones allowed for selection of retroviral integration based on GFP and mCherry fluorescent expression, respectively, and on puromycin-enriched medium. Plasmid DNA (pDNA) was extracted from bacterial cultures started from the glycerol stock of the constructs using a DNA Midiprep extraction kit (Qiagen, Hilden, Germany) following the manufacturer’s guidelines. The pDNA concentrations were measured by Nanodrop (ThermoFisher Scientific BVBA, Merelbeke, Belgium), and DNA quality cutoff values were set at a 260/230 ratio of 1.80 and a 280/230 ratio of 2. HEK293T cells were transfected using JetPEI reagents (Polyplus transfection, Illkirch, France) and the following plasmids: KD or OE vector (3 µg), a pCMV-VSV-G envelope (0.3 µg), and the packaging vector p8.91 (2.7 µg) in 250 µL NaCl. On the third day after transfection, the virus was harvested and concentrated by adding PEG-it virus precipitation solution (Sanbio, Uden, The Netherlands) and then centrifugating the mix after >12 h of incubation at 4 °C. Concentrated virus was aliquoted and stored at −80 °C. Leukemic cells were spin-infected in a centrifuge at 32 °C for 90 min at 2300 rpm, and puromycin selection (0.5 µg/mL) was initiated 24 h after transduction. After incubation with puromycin selection for 48 h, transduction efficiency was measured through GFP or mCherry fluorescence by flow cytometry (BD Lyrica, Aalst, Belgium), using the parental non-transduced cell lines (HL-60, KASUMI-1, THP-1, and MOLM-13) as controls. Puromycin selection was continued in case the transduction efficiency was less than 95%.

### 4.5. RNA Sequencing of Overexpression (OE) and Knockdown (KD) Models

Total RNA of the *CD68* OE HL-60 cell line (HL-60*^CD68^* ^OE^) and an empty vector control HL-60 cell line (HL-60^mock^), as well as the *CD68* KD THP1 cell line (THP-1*^CD68^* ^KD^), the NTC THP-1 cell line (THP-1^NTC^), were checked for purity and integrity on an electrophoresis gel. RNA sequencing was performed in triplicate for each cell line by Eurofins Genomics (Ebersberg, Germany). Sequencing was performed on a Genome Sequencer Illumina NovaSeq 6000 (S4 PE150 XP), producing 150 bp paired-end reads with a median library size of 32.7 million reads per sample. Fastq-files with paired-end data were aligned to the GRCh38.p14 reference sequence using STAR 2.7.6a. Read counts were determined using the featureCounts function of the R (v4.5.2) Subread package. Raw data are uploaded on the GEO platform and can be found under GSE287738. Differential gene expression analysis was performed independently for each model (HL-60 and KASUMI-1 CD68 overexpression; THP-1 and MOLM-13 CD68 knockdown) using the R DESeq2 package [51]. Genes with an adjusted *p*-value (Benjamini–Hochberg) < 0.05 were considered significantly differentially expressed within each dataset. The principal component analysis plots, volcano plots, and heatmaps were created in R (DESeq2 and Rtools packages; plotPCA, ComplexHeatmap, and EnhancedVolcano). Overlapping genes were defined as those significantly deregulated in at least one overexpression model and at least one knockdown model. To identify genes associated with CD68-dependent transcriptional programs, a directional filter was applied, requiring consistent bidirectional behavior across models, i.e., upregulation in at least one overexpression model and downregulation in at least one knockdown model, irrespective of statistical significance in the remaining models.

### 4.6. Functional Assays

A Cell Counting Kit 8 (WST-8) by Abcam (Cambridge, UK) was used to quantify cell proliferation. Briefly, 10,000 cells were plated per well in a clear-bottom 96-well plate. Immediately and after 24, 48, and 72 h, 10 µL of WST-8 Solution was added to each well. Cells were incubated for 4 h at 37 °C in the dark. Absorbance was measured at 460 nm on a Spectramax instrument (Molecular Devices, San Jose, CA, USA). For the cytotoxicity assay, 50,000 cells were plated per well in a clear-bottom 96-well plate. Different concentrations of cytarabine (0.001, 0.01, 0.1, 1, 10.0, 100.0 µM) were added to the cells, and incubated for 72 h in a 37 °C, 5% CO_2_ incubator. After 72 h, 10 µL of WST-8 Solution was also added to each well, incubated for 4 h at 37 °C in the dark, and absorbance was measured at 460 nm. For the cell cycle assay, 3.5 × 10^6^ cells of each sample were washed with ice-cold PBS, fixed with 70% ethanol, and incubated for at least 1 h at −20 °C. After incubation, cells were spun down, washed, and resuspended in cold PBS. RNase A (0.5 mg/mL) was added, and the cells were incubated for 1 h at 37 °C, 5% CO_2_, and 95% humidity. Propidium iodide (PI) for OE cell lines or DAPI for KD cell lines was added, and the cells were analyzed on a flow cytometry device (BD Fortessa, Aalst, Belgium). Sub-G1 and >G2 events were excluded from comparative cell cycle quantification. An example of the gating strategy can be found in Supplementary Methods Figure S15. For the apoptosis assay, 1 × 10^6^ cells were seeded for 48 h in a 24-well plate and were analyzed after addition of annexin V BV421 and 7-AAD on a flow cytometry device (BD Fortessa).

### 4.7. Gene Set Enrichment Analyses (GSEA)

Gene set enrichment analysis (GSEA) was performed using the pre-ranked option of the GSEA software (Broad Institute, version 4.2.3). For each model, genes were ranked based on a composite score calculated as the product of the log2 fold change and the negative logarithm of the adjusted *p*-value (−log10 adjusted *p*-value), thereby integrating both the magnitude and significance of differential expression. Pre-ranked gene lists were generated separately for each condition, as well as for averaged overexpression (HL-60 and KASUMI-1) and knockdown (THP-1 and MOLM-13) datasets. GSEA was conducted using the Molecular Signatures Database (MSigDB, version 2026), including the Hallmark (H) and curated pathway (C2:CP, Reactome/KEGG) gene sets. Default parameters were applied with 1000 gene set permutations, and gene sets with a false discovery rate (FDR) q-value < 0.25 were considered enriched, in line with standard GSEA guidelines. Figures of the enriched pathways were generated through Cytoscape software (version 3.10.4; https://cytoscape.org/).

### 4.8. Statistical Analysis

Univariate and multivariate Cox regression analyses were performed in R (survival and survminer packages) to assess the prognostic value of *CD68* expression for overall survival (OS) and event-free survival (EFS). CPM cutoffs for survival analyses were determined based on the optimal cutpoint (R package). Significant univariate factors were included in the multivariate model. Events were defined as death, induction failure, or relapse. OS and EFS probabilities of *CD68*-high versus *CD68*-low pedAML patients were estimated in GraphPad Prism 8 using Kaplan–Meier and log-rank tests. Differences in clinical characteristics, flow cytometry, Western blot, qPCR, and functional analyses were assessed with Mann–Whitney or Kruskal–Wallis tests in GraphPad Prism. Statistical significance was set at *p* < 0.05.

## Data Availability

All data generated or analyzed during this study are included in this published article (and its Supplementary Information Files). Raw RNA sequencing data have been uploaded to the GEO platform and can be found under GSE287738.

## Supplementary Materials

The following supporting information can be downloaded at: https://www.mdpi.com/article/10.3390/ijms27115136/s1.

## Abbreviations

The following abbreviations are used in this manuscript:

(ped)AML	(pediatric) acute myeloid leukemia
KD	knockdown
OE	overexpression
EFS	event-free survival
LSC	leukemic stem cell(s)
HSC	hematopoietic stem cell(s)
BM	bone marrow
TAM/LAM	tumor-associated macrophages/leukemia-associated macrophages
LAMP	lysosomal/endosomal-associated membrane glycoproteins
MRD	minimal residual disease
CB	cord blood
CPM	counts per million
GMP	granulocyte-monocyte progenitor
NTC	non-targeting shRNA
pDNA	plasmid DNA
PI	Propium iodide
GSEA	gene set enrichment analysis
FDR	false discovery rate
OS	overall survival
FC	fold change
MFI	median fluorescence intensity
LAIP	leukemia-associated immunophenotype
ELSc	embryonic-like stem cells
