# Supplementary material for: Exploring the Role of Macrophage Marker CD68 in Pediatric Acute Myeloid Leukemia
Source: Int J Mol Sci. 2026 Jun 5;27(11):5136. doi: 10.3390/ijms27115136 (PMC13257172; doi:10.3390/ijms27115136)
Supplement: Supplementary file 1 [file ijms-27-05136-s001.zip › ijms-4025563-supplementary.pdf]

Supplementary

Supplementary Table S1. Demographics of the TARGET patients with pedAML at diagnosis (n=1332).

	Mean (range)	
Age, years	9.75 (0-29)	
WBC count, x10^9 L	65.29 (0-918.5)	
Blast count		
BM, %	59.88 (0-100)*	
PB, %	44.08 (0-100)	
	**N**	**%**
Gender		
Male	694	52,1
Female	638	47,9
Occurence of event		
No	572	42,9
Yes	760	57,1
Death	86	6,5
Relapse	113	8,5
Induction Failure	561	42,1
Vital status		
Alive	844	63,4
Dead	488	36,6
Death	86	6,5
Death after induction failure	75	5,6
Death after relapse	327	24,5
Karyotype		
Normal	332	24,9
Abnormal	973	73,0
Unknown	27	2,0
Core-binding factor leukemia		
Yes	331	24,8
inv(16)	143	10,7
t(8;21)	188	14,1
No	974	73,1
Unknown	27	2,0
NPM1		
Mutated	115	8,6
Wild type	1207	90,6
Unknown	10	0,8
FLT3-ITD		
Positive	215	16,1
Wild type	1117	83,9
CEBPA		
Mutated	80	6,0
Wild type	1248	93,7
Unknown	4	0,3
WT1 overexpression		
Yes	26	2,0
No	260	19,5
Unknown	1046	78,5
CNS involvement		
Yes	1021	76,7
No	273	20,5
Unknown	38	2,9
Chloroma		
Yes	66	5,0
No	714	53,6
Unknown	552	41,4
Risk stratification		
High Risk (HR)	158	11,9
Standard Risk (SR)	661	49,6
Low Risk (LR)	491	36,9
Unknown	22	1,7
FAB classification		
M0	7	0,5
M1	37	2,8
M2	71	5,3
M4	66	5,0
M5	56	4,2
M6	6	0,5
M7	11	0,8
Unknown/not classified	1078	80,9
HSCT in first complete remission		
Yes	213	16,0
No	1033	77,6
Unknown	86	6,5

***one missing data; PedAML, pediatric acute myeloid leukemia; WBC, white blood cell; BM, bone marrow; PB, peripheral blood; FLT3-ITD, fms-like tyrosine kinase receptor-3 internal tandem duplication; NPM1, nucleophosmin; CEBPA, CCAAT/enhancer-binding protein alpha; FAB, French-British-American; WT1, Wilms' tumor 1; CNS, central nerve system; HR, high risk; SR, standard risk; LR, low risk; HSCT, hematopoietic stem cell transplantation.**

Supplementary Table S2. Expression, cytogenetic, molecular and clinical data of the pediatric acute myeloid leukemia (pedAML) samples of the TARGET database (n=1332).

Separate file

Supplementary Table S3. Fold change (FC) Median Fluorescence Intensity (MFI) of intracellular staining of CD68 in the bulk bone marrow mononuclear cells of patients with pedAML (n=8) and percentages of CD68 positive cells, analyzed by flow cytometry, ungated and gated by expression of the leukemia-associated immunophenotype (LAIP) markers.

	FC MFI leukemic blasts	% CD68+ leukemic blasts	FC MFI CD34+CD38- blasts
pedAML1	2.65	39.6	3.08
pedAML2	10.09	87.0	NaN
pedAML3	9.01	97.2	NaN
pedAML4	2.88	35.9	2.89
pedAML5	4.35	62.6	3.27
pedAML6	1.58	21.0	1.38
pedAML7	2.93	44.9	NaN
pedAML8	3.52	56.3	NaN

FC = fold change, MFI = median fluorescence intensity, + = positive , NaN = not analyzed due to too few or absent CD34+ cells.

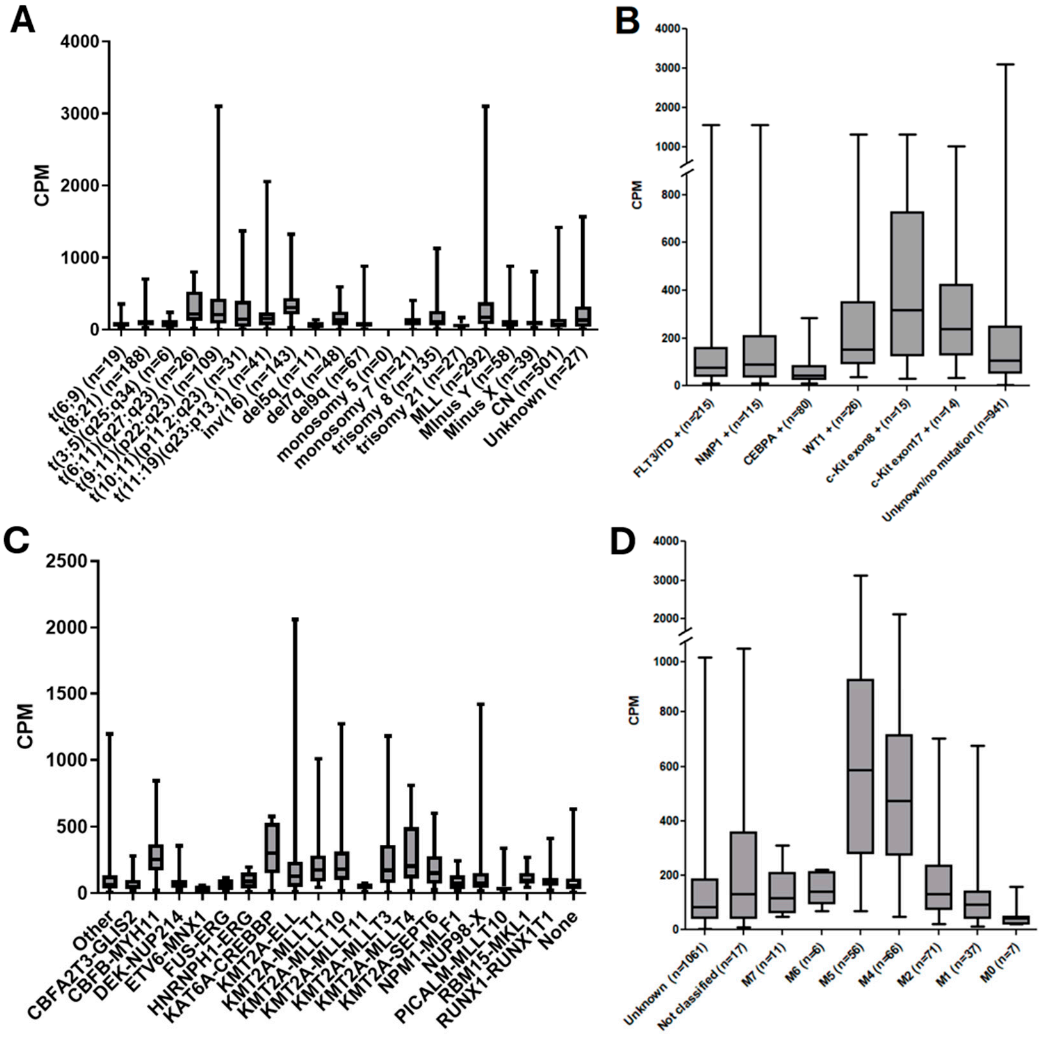

Supplementary Figure S1. *CD68* expression in CPM of pedAML samples of the TARGET database (n=1332) based on A) cytogenetics, B) molecular aberrations, C) fusions, and D) FAB classification.

GSE17855 DenBoer

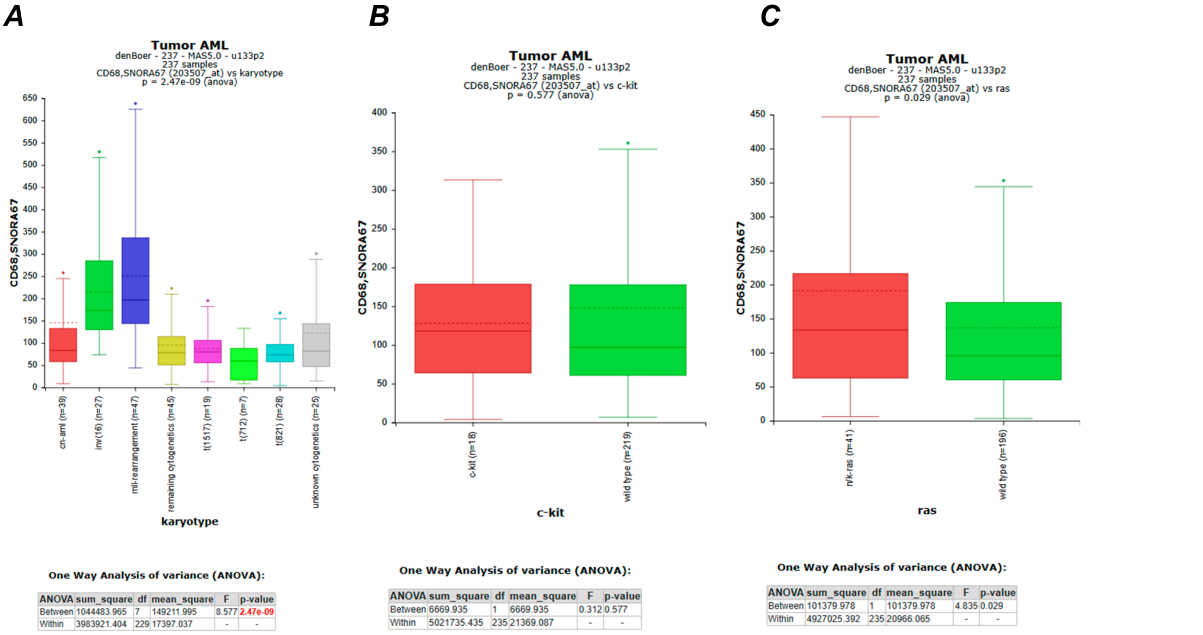

Supplementary Figure S2. Transcript expression of *CD68* in a pedAML cohort (n=237) of Den Boer et al, based on A) cytogenetic aberrations, B) presence of c-kit mutation, and C) presence of N/KRAS mutation. Figures generated through the R2 database.

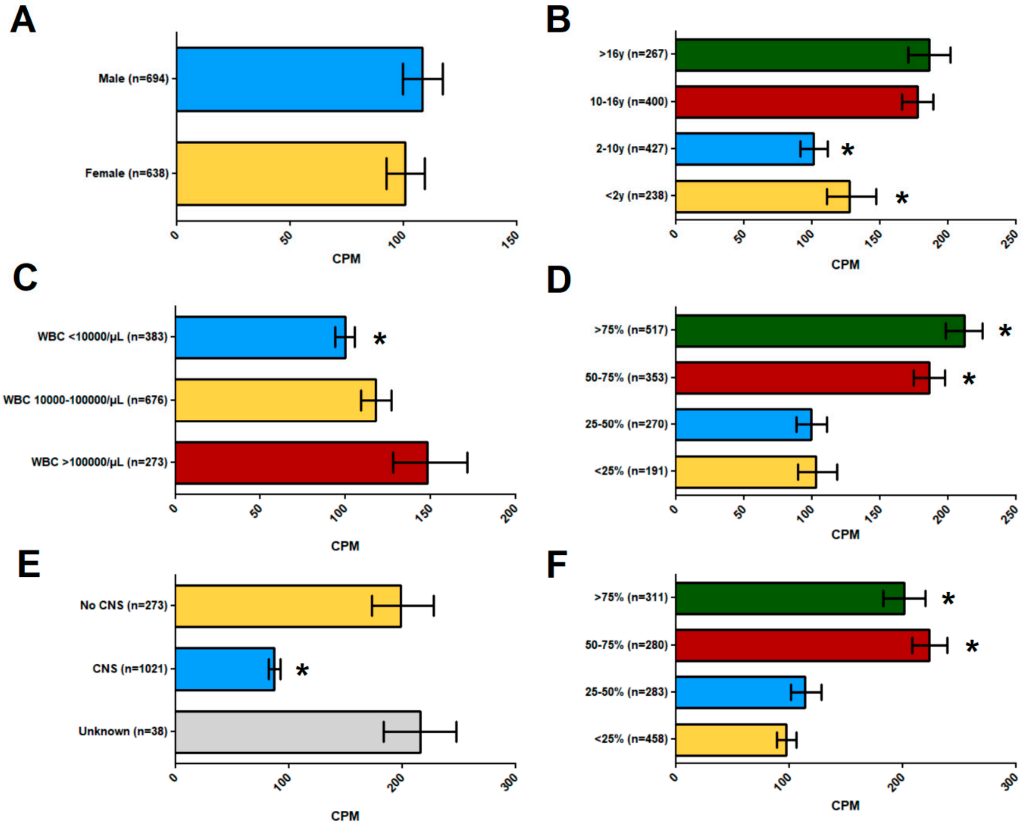

Supplementary Figure S3. *CD68* expression in counts per million (CPM) of pedAML samples of the TARGET database (n=1332) based on A) sex, B) age category, C) WBC count at diagnosis, D) blast percentage in the bone marrow at diagnosis, and E) central nervous system (CNS) invasion, F) blast percentage in the peripheral blood at diagnosis (* = p <0.05).

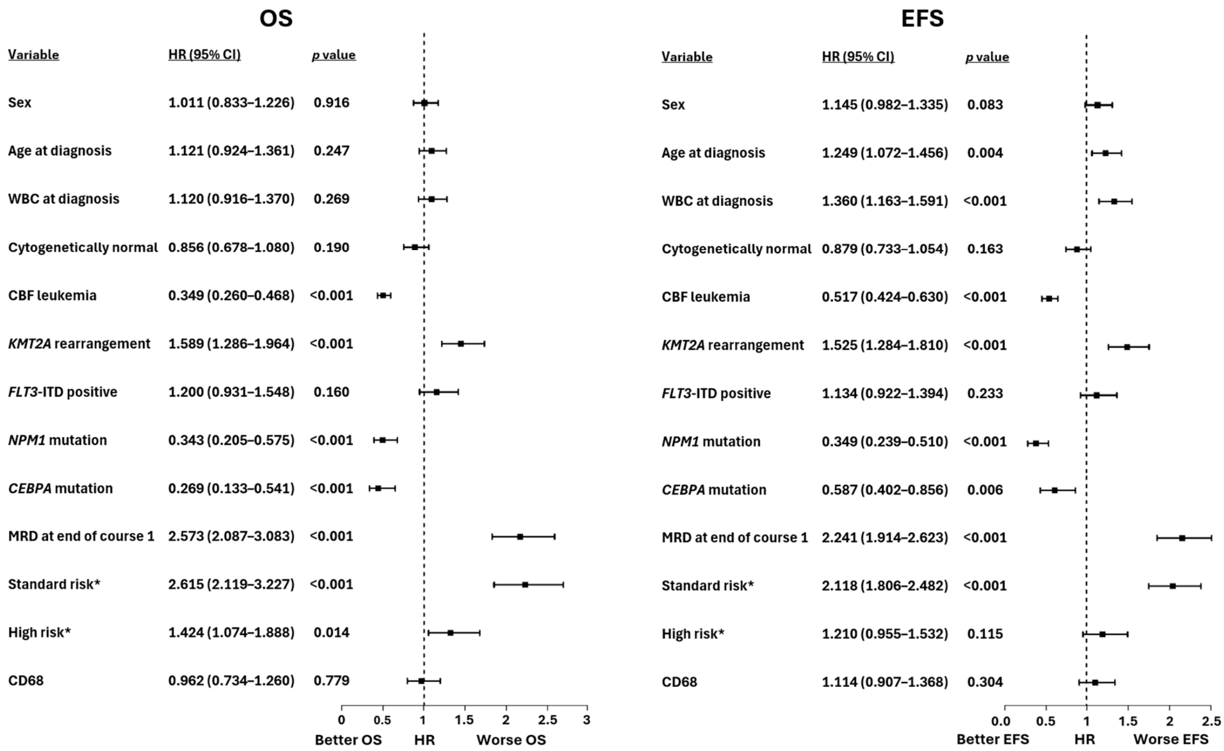

Supplementary Figure S4. Independent prognostic analysis of *CD68*. Forest plots of univariate independent Cox regression analyses of the overall survival (OS) and event-free survival (EFS) including *CD68* expression and other characteristics. *according to the COG protocols

Abbreviations: WBC, white blood cell; CBF, core-binding factor; MRD, minimal residual disease.

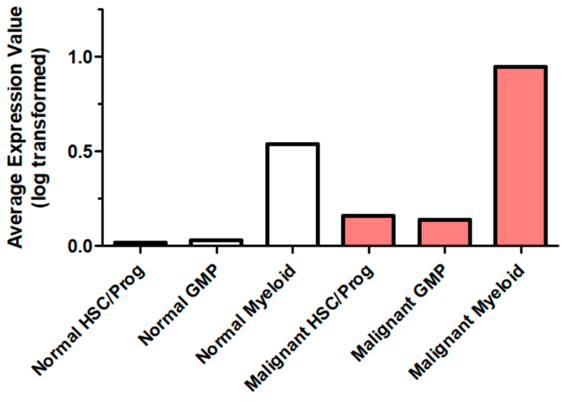

Supplementary Figure S5. Average expression values (log transformed) of *CD68* in different normal (white) and malignant (red) fractions from 16 patients with AML and 5 healthy donors. HSC/Prog = hematopoeietic stem cell or progenitor-like cells, GMP = granulocyte-macrophage progenitor cells.

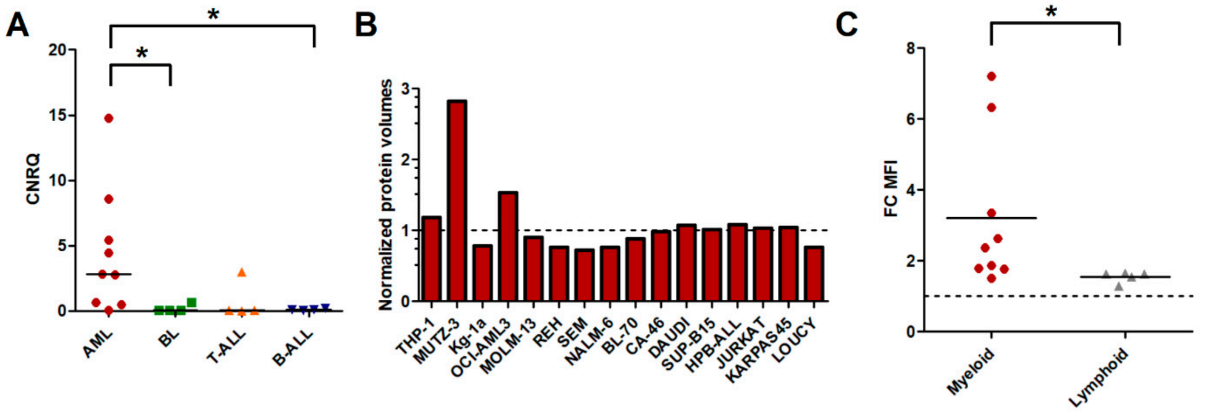

Supplementary Figure S6. A) Calibrated normalized relative quantity (CNRQ) values of *CD68* transcript levels on RT-qPCR in AML (n=9), Burkitt lymphoma (BL, n=4), T-ALL (n=4), and B-ALL (n=4) cell lines. B) Normalized expression of CD68 in hematological cell lines (n=15) on Western blot. C) FC MFI of intracellular staining of CD68 in myeloid cell lines (n=9) compared to lymphoid cell lines (n=5) on flow cytometry.

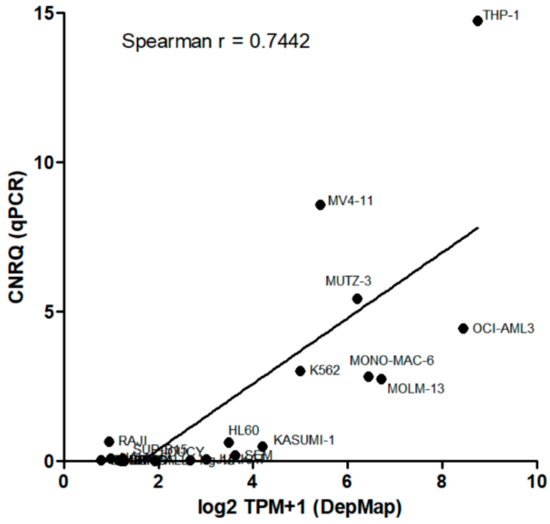

Supplementary Figure S7. Correlation plot of CNRQ values of CD68 on RT-qPCR compared to log2(TPM+1) values of the DepMap database.

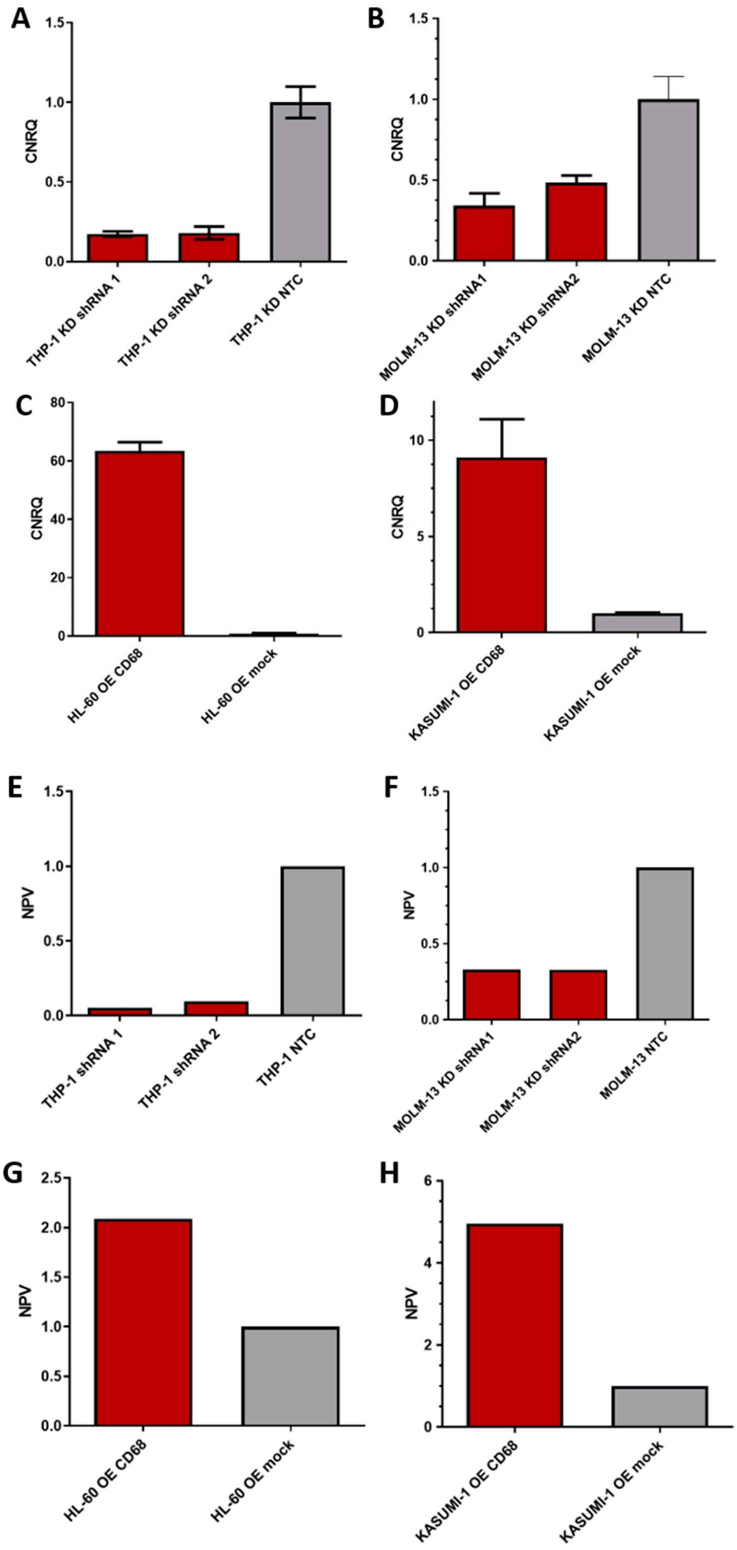

Supplementary Figure S8. A-D) Calibrated normalized relative quantity (CNRQ) values of human *CD68* measured by RT-qPCR in 2 THP-1$^{CD68\ KD}$ and THP-1^{NTC}, MOLM-13$^{CD68\ KD}$ and MOLM-13^{NTC}, HL-60$^{CD68\ \mathrm{OE}}$ and HL-60^{mock}, and KASUMI-1$^{CD68\ \mathrm{OE}}$ and KASUMI-1^{mock}. E-H) Normalized expression of CD68 in 3 THP-1$^{CD68\ KD}$ and THP-1^{NTC}, MOLM-13$^{CD68\ KD}$ and MOLM-13^{NTC}, HL-60$^{CD68\ \mathrm{OE}}$ and HL-60^{mock}, and KASUMI-1$^{CD68\ \mathrm{OE}}$ and KASUMI-1^{mock} on Western blot. RT-qPCR was performed with 3 technical replicates and normalized using two different house-keeping genes.

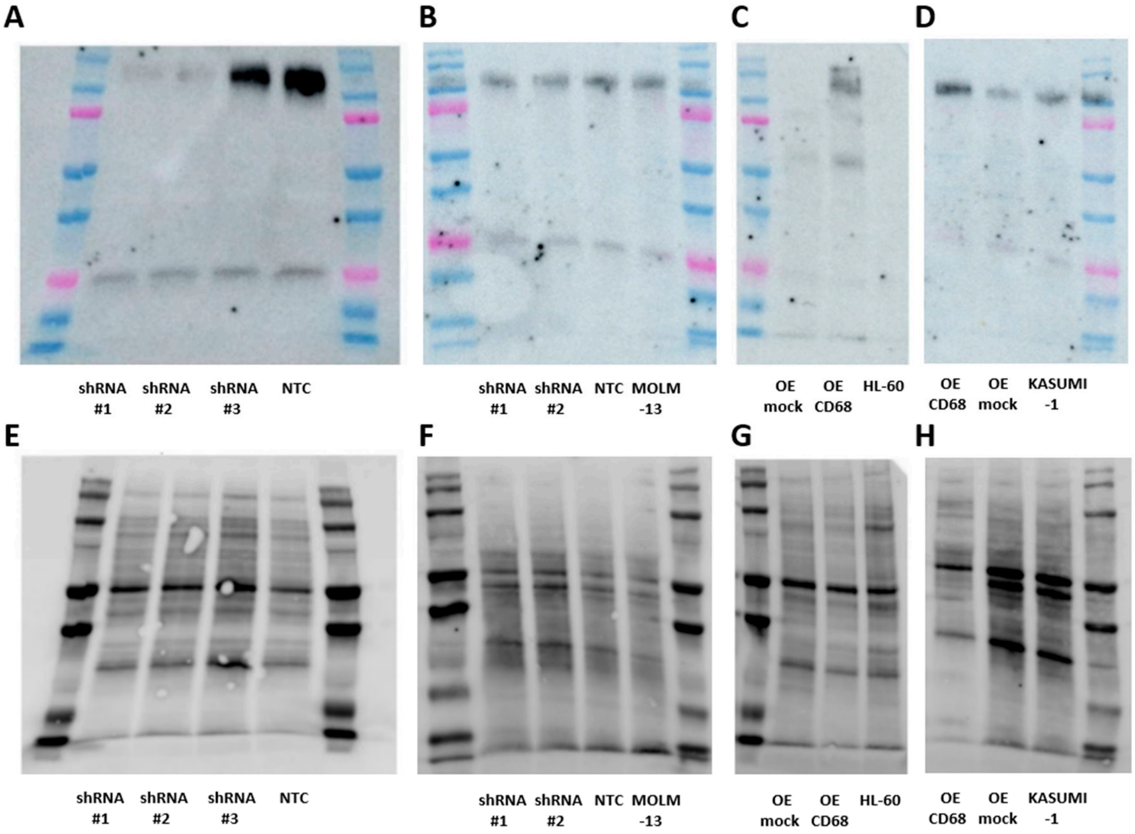

Supplementary Figure S9. Raw results of the Western blot analysis showing CD68 (A) and total protein (B) in 3 THP-1$^{CD68\ KD}$ compared to THP-1^{NTC}, CD68 (C) and total protein (D) in 2 MOLM-13$^{CD68\ KD}$ compared to MOLM-13^{NTC}, CD68 (E) and total protein (F) in HL-60$^{CD68\ OE}$ compared to HL-60^{mock}, and CD68 (G) and total protein (H) in KASUMI-1$^{CD68\ OE}$ compared to KASUMI-1^{mock}.

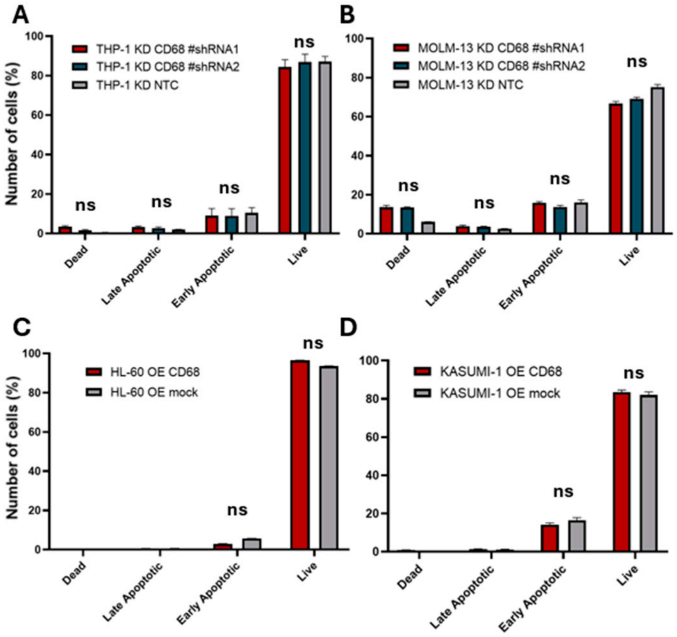

Supplementary Figure S10. Apoptosis assay of A) 2 THP-1$^{CD68\ KD}$, B) 2 MOLM-13$^{CD68\ KD}$, C) HL-60$^{CD68\ OE}$, and D) KASUMI-$^{1CD68\ OE}$ vs THP-1^{NTC}, MOLM-13^{NTC}, HL-60^{mock}, and KASUMI-1^{mock}, respectively. The assay was performed twice with each time three technical replicates.

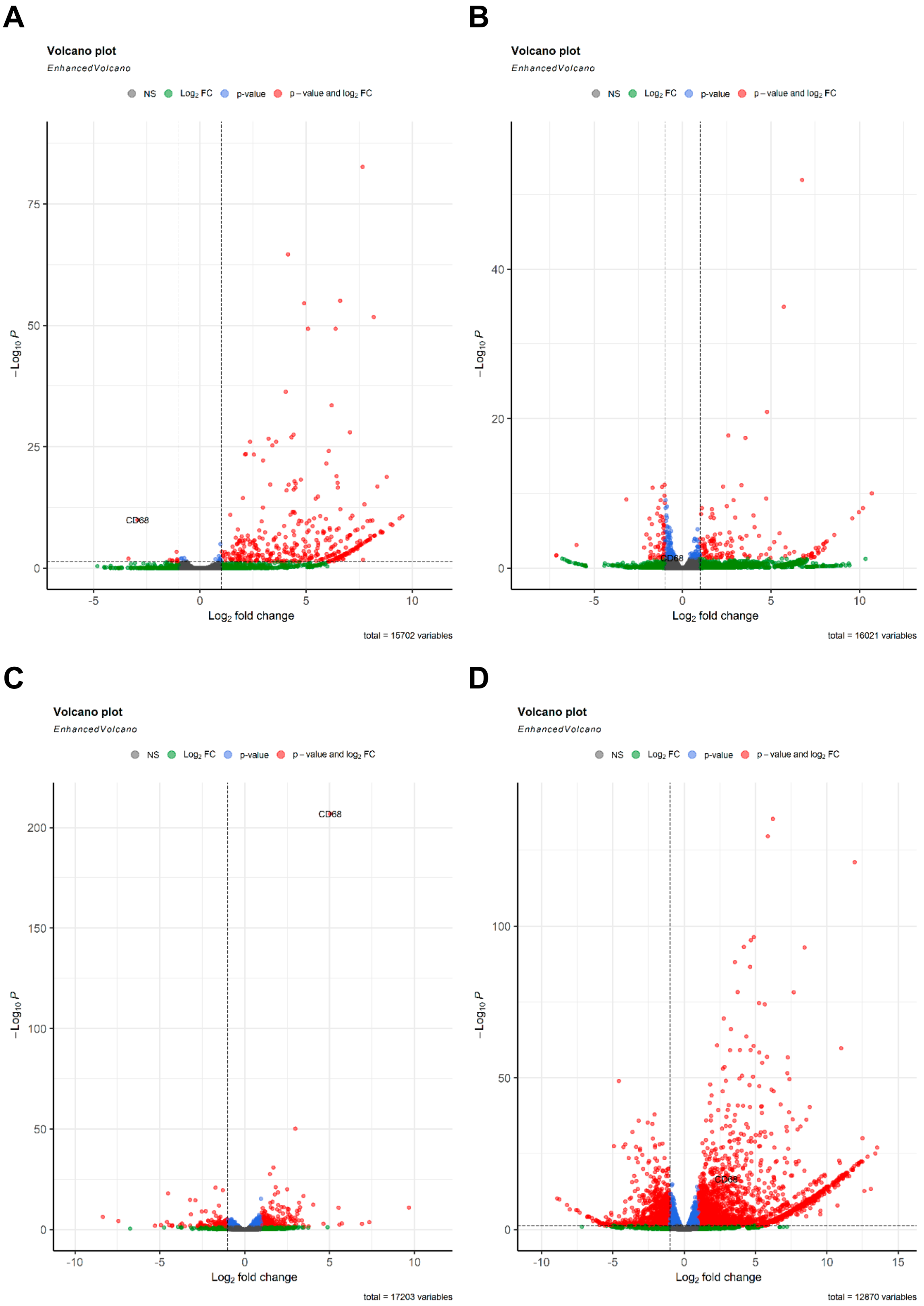

Supplementary Figure S11. Volcano plots of A) THP-1$^{CD68\ KD1}$ compared to THP-1^{NTC}, B) MOLM-13$^{CD68\ KD1}$ compared to MOLM-13^{NTC}, C) HL-60$^{CD68\ OE}$ compared to HL-60^{mock} , and D) KASUMI-1$^{CD68\ OE}$ compared to KASUMI-1^{mock}.

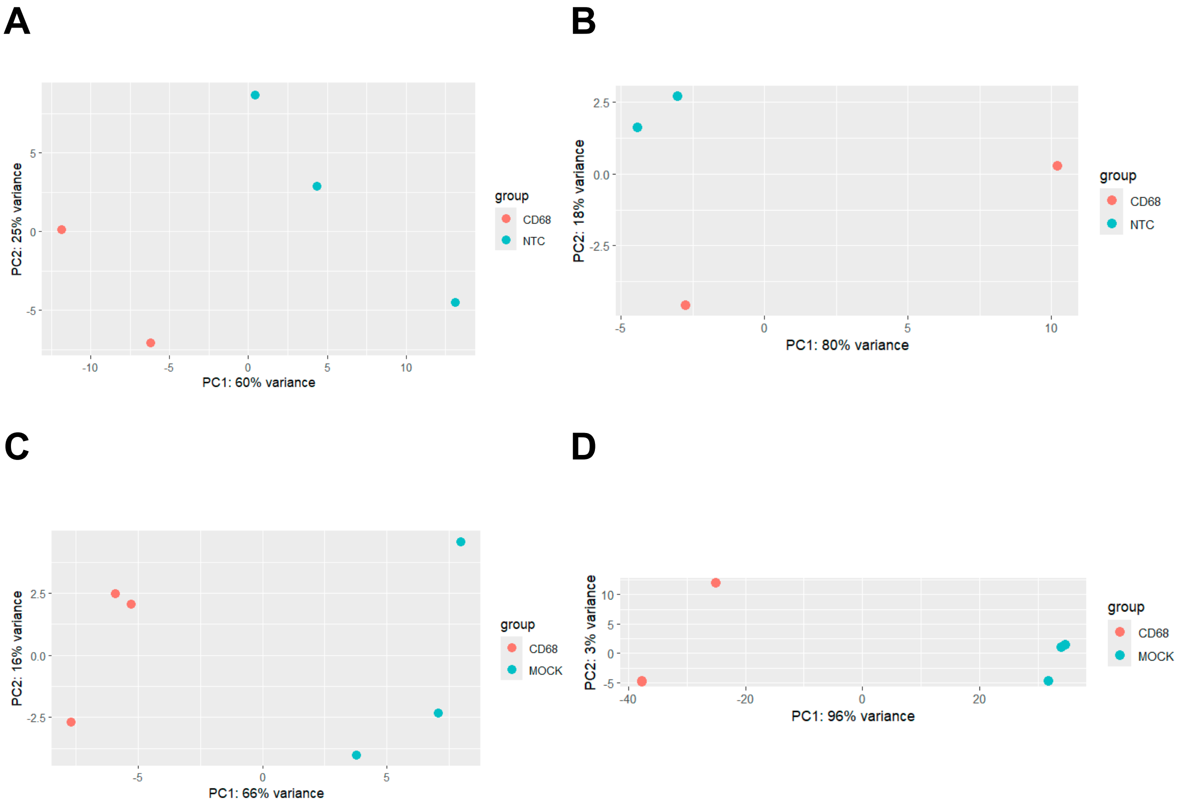

Supplementary Figure S12. Principal component analysis (PCA) plots of A) THP-1$^{CD68\ KD1}$ compared to THP-1^{NTC}, B) MOLM-13$^{CD68\ KD1}$ compared to MOLM-13^{NTC}, C) HL-60$^{CD68\ OE}$ compared to HL-60^{mock} , and D) KASUMI-1$^{CD68\ OE}$ compared to KASUMI-1^{mock}.

Supplementary Table S4. Overview of the enriched Hallmark and C2-CP pathways with an FDR<25% and <10%, respectively, of all significantly up- and downregulated genes of both overexpression models compared to their respective mocks.

**Upregulated Hallmark Pathways**	**nGenes**	**NES**	**NOM p-value**	**FDR q-value**
HALLMARK_TGF_BETA_SIGNALING	47	1.52	0.016	0.056
HALLMARK_NOTCH_SIGNALING	30	1.46	0.039	0.080
**Upregulated C2-CP Pathways**				
WP_STEROL_REGULATORY_ELEMENTBINDING_PROTEINS_SREBP_SIGNALING	64	1.87	0.000	0.000
REACTOME_HOST_INTERACTIONS_OF_HIV_FACTORS	111	1.84	0.000	0.000
REACTOME_MAPK6_MAPK4_SIGNALING	69	1.81	0.000	0.000
REACTOME_REGULATION_OF_HOMOTYPIC_CELL_CELL_ADHESION	137	1.81	0.000	0.000
REACTOME_GOLGI_TO_ER_RETROGRADE_TRANSPORT	119	1.80	0.000	0.000
REACTOME_NEGATIVE_REGULATION_OF_MAPK_PATHWAY	40	1.77	0.000	0.002
WP_NF1_COPY_NUMBER_VARIATION_SYNDROME	68	1.77	0.000	0.002
REACTOME_COPI_DEPENDENT_GOLGI_TO_ER_RETROGRADE_TRAFFIC	87	1.76	0.000	0.002
REACTOME_RNA_POLYMERASE_II_TRANSCRIBES_SNRNA_GENES	78	1.76	0.000	0.002
PID_PLK1_PATHWAY	45	1.74	0.000	0.004
REACTOME_REGULATION_OF_PTEN_STABILITY_AND_ACTIVITY	51	1.73	0.000	0.006
WP_FANCONI_ANEMIA	46	1.72	0.000	0.007
WP_HEAD_AND_NECK_SQUAMOUS_CELL_CARCINOMA	65	1.72	0.000	0.007
PID_ERBB1_DOWNSTREAM_PATHWAY	97	1.71	0.000	0.006
REACTOME_CELLULAR_RESPONSE_TO_HEAT_STRESS	88	1.70	0.003	0.011
WP_RNAPOLYMERASE_II_TRANSCRIPTION_THE_PREINITIATION_COMPLEX	64	1.69	0.000	0.011

PID_PI3KCI_PATHWAY	43	1.69	0.006	0.011
WP_SMITHMAGENIS_AND_POTOCKILUPSKI_SYNDROME_COPY_NUMBER_VARIATION	132	1.69	0.000	0.013
WP_TH17_CELL_DIFFERENTIATION_PATHWAY	52	1.68	0.000	0.014
REACTOME_DEADENYLATION_DEPENDENT_MRNA_DECAY	46	1.68	0.003	0.014
BIOCARTA_TCR_PATHWAY	41	1.66	0.003	0.021
WP_MELANOMA	58	1.66	0.006	0.020
WP_SMALL_CELL_LUNG_CANCER	82	1.66	0.000	0.020
KEGG_ADIPOCYTOKINE_SIGNALING_PATHWAY	48	1.66	0.009	0.020
WP_CYTOSOLIC_DNASENSING_PATHWAY	49	1.66	0.000	0.020
REACTOME_FLT3_SIGNALING	35	1.66	0.000	0.019
REACTOME_REGULATION_OF_RAS_BY_GAPS	50	1.66	0.000	0.019
PID_IL2_1PATHWAY	51	1.65	0.009	0.020
WP_INTERFERON_TYPE_I_SIGNALING	52	1.64	0.004	0.024
KEGG_MEDICUS_REFERENCE_COPI_VESICLE_FORMATION	23	1.63	0.000	0.034
WP_IL2_SIGNALING	38	1.63	0.000	0.033
KEGG_PROTEASOME	36	1.62	0.003	0.033
PID_AR_TF_PATHWAY	44	1.62	0.000	0.037
REACTOME_RHOD_GTPASE_CYCLE	49	1.62	0.000	0.037
PID_IL2_PI3K_PATHWAY	32	1.61	0.000	0.039
REACTOME_TRANSCRIPTIONAL_REGULATION_OF_WHITE_ADIPOCYTE_DIFFERENTIATION	72	1.61	0.003	0.042
REACTOME_NOD1_2_SIGNALING_PATHWAY	36	1.60	0.006	0.047
REACTOME_ACTIVATION_OF_ANTERIOR_HOX_GENES_IN_HINDBRAIN_DEVELOPMENT_DURING_EARLY_EMBRYOGENESIS	56	1.60	0.006	0.051
WP_HOSTPATHOGEN_INTERACTION_OF_HUMAN_CORONAVIRUSES_MAPK_SIGNALING	34	1.59	0.000	0.053
REACTOME_RAF_ACTIVATION	31	1.59	0.003	0.055
KEGG_SMALL_CELL_LUNG_CANCER	74	1.59	0.000	0.054
PID_IFNG_PATHWAY	38	1.59	0.006	0.053
PID_P38_ALPHA_BETA_PATHWAY	25	1.59	0.000	0.057
KEGG_BASE_EXCISION_REPAIR	32	1.58	0.000	0.065
REACTOME_BASE_EXCISION_REPAIR	41	1.58	0.009	0.064
WP_P53_TRANSCRIPTIONAL_GENE_NETWORK	78	1.57	0.013	0.073
REACTOME_TRANSCRIPTIONAL_REGULATION_BY_MECP2	45	1.57	0.017	0.072
REACTOME_RHOH_GTPASE_CYCLE	34	1.57	0.005	0.074
REACTOME_INTERLEUKIN_17_SIGNALING	66	1.57	0.006	0.075
PID_TXA2PATHWAY	46	1.57	0.014	0.075
WP_MAPK_SIGNALING_AND_ARTD_FAMILY_MEMBERS	32	1.56	0.013	0.076
WP_BASE_EXCISION_REPAIR	29	1.56	0.007	0.077
WP_GASTRIN_SIGNALING	98	1.56	0.000	0.079
WP_CARDIAC_HYPERTROPHIC_RESPONSE	46	1.56	0.006	0.079
REACTOME_PTEN_REGULATION	117	1.56	0.000	0.078
KEGG_CYTOSOLIC_DNA_SENSING_PATHWAY	31	1.56	0.008	0.081
KEGG_INOSITOL_PHOSPHATE_METABOLISM	50	1.56	0.013	0.081
WP_ANDROGEN_RECEPTOR_SIGNALING	84	1.55	0.003	0.086
REACTOME_SUMOYLATION_OF_CHROMATIN_ORGANIZATION_PROTEINS	52	1.55	0.017	0.085
REACTOME_RESOLUTION_OF_ABASIC_SITES_AP_SITES	35	1.55	0.011	0.084
SIG_PIP3_SIGNALING_IN_CARDIAC_MYOCTES	55	1.55	0.006	0.085
REACTOME_GENE_AND_PROTEIN_EXPRESSION_BY_JAK_STAT_SIGNALING_AFTER_IL12_STIMULATION	31	1.54	0.037	0.099
**Downregulated Hallmark Pathways**				
HALLMARK_MYC_TARGETS_V2	55	-1.65	0.000	0.000
HALLMARK_UNFOLDED_PROTEIN_RESPONSE	105	-1.64	0.000	0.000
HALLMARK_G2M_CHECKPOINT	188	-1.60	0.000	0.000
HALLMARK_REACTIVE_OXYGEN_SPECIES_PATHWAY	46	-1.36	0.067	0.204
HALLMARK_APOPTOSIS	142	-1.33	0.020	0.225
HALLMARK_KRAS_SIGNALING_DN	102	-1.30	0.041	0.232
**Downregulated C2-CP Pathways**				
REACTOME_ACTIVATION_OF_NF_KAPPAB_IN_B_CELLS	51	-1.75	0.000	0.000
REACTOME_DOWNSTREAM_SIGNALING_EVENTS_OF_B_CELL_RECEPTOR_BCR	64	-1.74	0.000	0.000
REACTOME_COMPLEX_I_BIOGENESIS	59	-1.72	0.000	0.000
REACTOME_FORMATION_OF_PARAXIAL_MESODERM	49	-1.70	0.000	0.000
REACTOME_CELLULAR_RESPONSE_TO_CHEMICAL_STRESS	169	-1.68	0.000	0.000

REACTOME_DEGRADATION_OF_BETA_CATENIN_BY_THE_DESTRUCTION_COMPLEX	68	-1.68	0.000	0.000
WP_OXIDATIVE_PHOSPHORYLATION	47	-1.66	0.000	0.000
KEGG_MEDICUS_VARIANT_MUTATION_CAUSED_ABERRANT_ABETA_TO_ELECTRON_TRANSFER_IN_COMPLEX_I	41	-1.66	0.000	0.000
REACTOME_ABC_TRANSPORTER_DISORDERS	58	-1.65	0.000	0.000
WP_MITOCHONDRIAL_COMPLEX_I_ASSEMBLY_MODEL_OXPHOS_SYSTEM	47	-1.65	0.000	0.000
REACTOME_SCF_SKP2_MEDIATED_DEGRADATION_OF_P27_P21	48	-1.64	0.000	0.001
KEGG_B_CELL_RECEPTOR_SIGNALING_PATHWAY	67	-1.63	0.000	0.001
REACTOME_SOMITOGENESIS	38	-1.63	0.000	0.001
REACTOME_DUAL_INCISION_IN_GG_NER	38	-1.63	0.000	0.001
KEGG_MEDICUS_VARIANT_MUTATION_CAUSED_ABERRANT_SNCA_TO_ELECTRON_TRANSFER_IN_COMPLEX_I	36	-1.62	0.000	0.002
REACTOME_RRNA_MODIFICATION_IN_THE_NUCLEUS_AND_CYTOSOL	55	-1.62	0.000	0.002
REACTOME_FCERI_MEDIATED_NF_KB_ACTIVATION	67	-1.61	0.000	0.003
KEGG_MEDICUS_VARIANT_MUTATION_CAUSED_ABERRANT_TDP43_TO_ELECTRON_TRANSFER_IN_COMPLEX_I	36	-1.61	0.000	0.003
REACTOME_IRE1ALPHA_ACTIVATES_CHAPERONES	48	-1.60	0.000	0.004
PID_CXCR3_PATHWAY	38	-1.60	0.000	0.004
KEGG_MEDICUS_REFERENCE_ELECTRON_TRANSFER_IN_COMPLEX_I	35	-1.60	0.000	0.005
REACTOME_CYTOPROTECTION_BY_HMOX1	50	-1.59	0.002	0.007
KEGG_ALZHEIMERS_DISEASE	136	-1.58	0.000	0.009
WP_TRANSCRIPTION_ACTIVATION_RNAPOLYMERASE_I_AND_KAT2AB_AND_INHIBITION_NURD_COMPLEX	37	-1.58	0.000	0.009
REACTOME_REGULATION_OF_GLUCOKINASE_BY_GLUCOKINASE_REGULATORY_PROTEIN	29	-1.58	0.000	0.010
KEGG_MEDICUS_VARIANT_MUTATION_INACTIVATED_PINK1_TO_ELECTRON_TRANSFER_IN_COMPLEX_I	36	-1.58	0.000	0.010
PID_P73PATHWAY	69	-1.57	0.000	0.010
WP_16P122_COPY_NUMBER_VARIATION_SYNDROME_520KB	45	-1.57	0.000	0.010
REACTOME_FANCONI_ANEMIA_PATHWAY	39	-1.57	0.000	0.010
REACTOME_HEME_SIGNALING	44	-1.57	0.000	0.010
REACTOME_GAMMA_CARBOXYLATION_HYPUSINYLATION_HYDROXYLATION_AND_ARYLSULFATASE_ACTIVATION	54	-1.57	0.000	0.010
PID_TRKR_PATHWAY	51	-1.57	0.000	0.010
KEGG_ACUTE_MYELOID_LEUKEMIA	52	-1.57	0.000	0.011
REACTOME_MITOCHONDRIAL_PROTEIN_DEGRADATION	83	-1.57	0.000	0.012
REACTOME_CLEC7A_DECTIN_1_SIGNALING	83	-1.56	0.001	0.013
WP_RETINOBLASTOMA_GENE_IN_CANCER	86	-1.56	0.000	0.013
PID_REG_GR_PATHWAY	62	-1.56	0.000	0.013
REACTOME_RIBOSOME_QUALITY_CONTROL_RQC_COMPLEX_EXTRACTS_AND_DEGRADES_NASCENT_PEPTIDE	98	-1.56	0.003	0.015
PID_ERBB2_ERBB3_PATHWAY	38	-1.56	0.000	0.015
WP_10Q1121Q1123_COPY_NUMBER_VARIATION_SYNDROME	46	-1.56	0.000	0.015
REACTOME_DEACTIVATION_OF_THE_BETA_CATENIN_TRANSACTIVATING_COMPLEX	38	-1.55	0.000	0.018
REACTOME_ATP_DEPENDENT_CHROMATIN_REMODELERS	28	-1.55	0.002	0.021
REACTOME_FC_EPSILON_RECEPTOR_FCERI_SIGNALING	114	-1.54	0.001	0.022
REACTOME_REGULATION_OF_PD_L1_CD274_POST_TRANSLATIONAL_MODIFICATION	75	-1.53	0.003	0.028
KEGG_MEDICUS_REFERENCE_CORE_NER_REACTION	27	-1.53	0.000	0.029
REACTOME_NS1_MEDIATED_EFFECTS_ON_HOST_PATHWAYS	40	-1.53	0.000	0.033
WP_ONCOSTATIN_M_SIGNALING	58	-1.53	0.013	0.032
REACTOME_RND2_GTPASE_CYCLE	35	-1.53	0.003	0.033
WP_ANGIOGENESIS_OVERVIEW	53	-1.53	0.001	0.033
WP_HEMATOPOIETIC_STEM_CELL_DIFFERENTIATION	47	-1.52	0.003	0.039
WP_NONALCOHOLIC_FATTY_LIVER_DISEASE	129	-1.52	0.001	0.039
REACTOME_SIGNALLING_TO_ERKS	29	-1.52	0.002	0.040
REACTOME_SIGNALING_BY_FGFR2	57	-1.51	0.006	0.048
WP_IL19_SIGNALING	22	-1.51	0.000	0.051
REACTOME_CHROMATIN_ORGANIZATION	202	-1.51	0.001	0.052
WP_NEUROGENESIS_REGULATION_IN_THE_OLFACTORY_EPITHELIUM	44	-1.50	0.009	0.052
REACTOME_CILIUM_ASSEMBLY	172	-1.50	0.000	0.053
REACTOME_RETROGRADE_TRANSPORT_AT_THE_TRANS_GOLGI_NETWORK	46	-1.50	0.012	0.052
REACTOME_FORMATION_OF_NEURONAL_PROGENITOR_AND_NEURONAL_BAF_NPBAF_AND_NBAF	22	-1.50	0.005	0.052
REACTOME_HH_MUTANTS_ABROGATE_LIGAND_SECRETION	42	-1.50	0.012	0.053
REACTOME_COMPLEX_IV_ASSEMBLY	38	-1.50	0.010	0.053
REACTOME_LATE_SARS_COV_2_INFECTION_EVENTS	65	-1.50	0.004	0.059
REACTOME_CARGO_TRAFFICKING_TO_THE_PERICILIARY_MEMBRANE	62	-1.49	0.000	0.061
REACTOME_SIGNALING_BY_THE_B_CELL_RECEPTOR_BCR	94	-1.49	0.000	0.060

REACTOME_ORGANELLE_BIOGENESIS_AND_MAINTENANCE	253	-1.49	0.000	0.062
WP_VITAMIN_B12_METABOLISM	39	-1.49	0.009	0.066
KEGG_COLORECTAL_CANCER	59	-1.49	0.009	0.070
WP_EFFECT_OF_PROGERIN_ON_GENES_INVOLVED_IN_PROGERIA	22	-1.48	0.000	0.077
REACTOME_GSK3B_MEDIATED_PROTEASOMAL_DEGRADATION_OF_PD_L1_CD274	42	-1.48	0.005	0.077
REACTOME_HDR_THROUGH_HOMOLOGOUS_RECOMBINATION_HRR	68	-1.48	0.008	0.081
WP_PERTURBATIONS_TO_HOSTCELL_AUTOPHAGY_INDUCED_BY_SARSCOV2_PROTEINS	39	-1.48	0.009	0.080
REACTOME_TGF_BETA_RECEPTOR_SIGNALING_ACTIVATES_SMADS	43	-1.47	0.016	0.086
WP_BARDETBIEDL_SYNDROME	75	-1.47	0.006	0.088
REACTOME_SPOP_MEDIATED_PROTEASOMAL_DEGRADATION_OF_PD_L1_CD274	43	-1.47	0.000	0.089
REACTOME_SIGNALING_BY_TGFB_FAMILY_MEMBERS	139	-1.47	0.005	0.091
REACTOME_TRAF6_MEDIATED_NF_KB_ACTIVATION	20	-1.47	0.012	0.099
REACTOME_VXPX_CARGO_TARGETING_TO_CILIUM	20	-1.47	0.003	0.098
REACTOME_FORMATION_OF_THE_BETA_CATENIN_TCF_TRANSACTIVATING_COMPLEX	32	-1.47	0.005	0.099
WP_JAKSTAT_SIGNALING_IN_THE_REGULATION_OF_BETA_CELLS	29	-1.46	0.012	0.099
REACTOME_SARS_COV_1_ACTIVATES_MODULATES_INNATE_IMMUNE_RESPONSES	38	-1.46	0.003	0.099
REACTOME_COPII_MEDIATED_VESICLE_TRANSPORT	66	-1.46	0.012	0.099

nGenes = number of genes, NES = normalized enrichment score, NOM = nominal, FDR = false discovery rate.

Supplementary Table S5. Overview of the enriched Hallmark and C2-CP pathways with an FDR<25% and <10%, respectively, of all significantly up- and downregulated genes of both knockdown models compared to their respective NTCs.

**Upregulated Hallmark Pathways**	**nGenes**	**NES**	**NOM p-value**	**FDR q-value**
HALLMARK_OXIDATIVE_PHOSPHORYLATION	199	1.45	0.000	0.000
HALLMARK_MITOTIC_SPINDLE	195	1.45	0.000	0.000
HALLMARK_MTORC1_SIGNALING	196	1.44	0.000	0.000
HALLMARK_E2F_TARGETS	197	1.41	0.000	0.001
HALLMARK_PROTEIN_SECRETION	93	1.37	0.000	0.021
HALLMARK_HYPOXIA	184	1.36	0.008	0.039
HALLMARK_MYC_TARGETS_V1	195	1.35	0.012	0.039
HALLMARK_PI3K_AKT_MTOR_SIGNALING	97	1.35	0.000	0.035
HALLMARK_HEME_METABOLISM	172	1.35	0.014	0.044
HALLMARK_EPITHELIAL_MESENCHYMAL_TRANSITION	169	1.31	0.029	0.100
HALLMARK_INTERFERON_ALPHA_RESPONSE	94	1.31	0.001	0.102
HALLMARK_TNFA_SIGNALING_VIA_NFKB	193	1.29	0.050	0.131
HALLMARK_ADIPOGENESIS	192	1.28	0.054	0.147
HALLMARK_UV_RESPONSE_UP	147	1.28	0.079	0.151
HALLMARK_IL2_STAT5_SIGNALING	185	1.27	0.062	0.157
HALLMARK_UV_RESPONSE_DN	134	1.25	0.114	0.208
HALLMARK_ALLOGRAFT_REJECTION	160	1.24	0.098	0.242
**Upregulated C2-CP Pathways**				
REACTOME_TRANSCRIPTIONAL_REGULATION_BY_TP53	348	1.55	0.000	0.000
REACTOME_CLASS_I_MHC_MEDIATED_ANTIGEN_PROCESSING_PRESENTATION	359	1.48	0.000	0.000
REACTOME_HIV_INFECTION	212	1.47	0.000	0.000
REACTOME_AEROBIC_RESPIRATION_AND_RESPIRATORY_ELECTRON_TRANSPORT	235	1.45	0.000	0.000
REACTOME_ANTIGEN_PROCESSING_UBIQUITINATION_PROTEASOME_DEGRADATION	284	1.45	0.000	0.000
KEGG_MAPK_SIGNALING_PATHWAY	227	1.43	0.000	0.000
REACTOME_HIV_LIFE_CYCLE	146	1.43	0.000	0.001
REACTOME_RESPIRATORY_ELECTRON_TRANSPORT	143	1.43	0.000	0.000
WP_MAPK_SIGNALING	213	1.43	0.000	0.001
WP_VEGFAVEGFR2_SIGNALING	418	1.42	0.000	0.001
REACTOME_NEDDYLATION	221	1.42	0.000	0.001
WP_CIRCADIAN_RHYTHM_GENES	158	1.42	0.000	0.001
REACTOME_REGULATION_OF_T_CELL_ACTIVATION_BY_CD28_FAMILY	194	1.42	0.000	0.001
REACTOME_CELL_CYCLE_CHECKPOINTS	254	1.42	0.000	0.001
REACTOME_DENGUE_VIRUS_HOST_INTERACTIONS	224	1.42	0.000	0.001

REACTOME_EPIGENETIC_REGULATION_OF_GENE_EXPRESSION	250	1.41	0.000	0.001
REACTOME_TOLL_LIKE_RECEPTOR_CASCADES	165	1.40	0.000	0.002
REACTOME_DEATH_RECEPTOR_SIGNALING	151	1.39	0.000	0.005
KEGG_OXIDATIVE_PHOSPHORYLATION	108	1.39	0.000	0.005
REACTOME_CO_INHIBITION_BY_PD_1	146	1.39	0.000	0.006
WP_PLEURAL_MESOTHELIOMA	377	1.39	0.002	0.006
WP_BDNF_SIGNALING_IN_NEURODEVELOPMENT	118	1.38	0.000	0.006
REACTOME_HOST_INTERACTIONS_OF_HIV_FACTORS	113	1.38	0.000	0.006
WP_DNA_DAMAGE_RESPONSE_ONLY_ATM_DEPENDENT	99	1.38	0.000	0.006
REACTOME_REGULATION_OF_PD_L1_CD274_EXPRESSION	132	1.38	0.000	0.007
WP_NONALCOHOLIC_FATTY_LIVER_DISEASE	139	1.38	0.000	0.007
WP_RAS_SIGNALING	163	1.38	0.002	0.007
REACTOME_MITOCHONDRIAL_TRANSLATION	97	1.38	0.000	0.009
KEGG_PARKINSONS_DISEASE	106	1.37	0.000	0.014
REACTOME_CDC42_GTPASE_CYCLE	150	1.37	0.001	0.014
PID_ERBB1_DOWNSTREAM_PATHWAY	103	1.36	0.000	0.015
WP_ELECTRON_TRANSPORT_CHAIN_OXPHOS_SYSTEM_IN_MITOCHONDRIA	91	1.36	0.000	0.015
WP_RETINOBLASTOMA_GENE_IN_CANCER	87	1.36	0.000	0.019
REACTOME_TOLL_LIKE_RECEPTOR_TLR1_TLR2_CASCADE	112	1.36	0.000	0.024
REACTOME_METABOLISM_OF_VITAMINS_AND_COFACTORS	167	1.35	0.003	0.028
WP_CAMKK2_PATHWAY	98	1.35	0.000	0.032
WP_REGULATION_OF_ACTIN_CYTOSKELETON	127	1.35	0.004	0.032
KEGG_ALZHEIMERS_DISEASE	146	1.35	0.012	0.031
KEGG_SPLICEOSOME	125	1.35	0.001	0.032
REACTOME_TCR_SIGNALING	96	1.34	0.001	0.037
WP_EFFECT_OF_OMEGA3_PUFA_ON_HUNTINGTONS_DISEASE_PATHWAYS	106	1.34	0.004	0.037
WP_BRAINDERIVED_NEUROTROPHIC_FACTOR_BDNF_SIGNALING	134	1.34	0.007	0.038
WP_FRAGILE_X_SYNDROME	108	1.34	0.001	0.038
REACTOME_EPIGENETIC_REGULATION_BY_WDR5_CONTAINING_HISTONE_MODIFYING_COMPLEXES	104	1.34	0.000	0.039
REACTOME_TOLL_LIKE_RECEPTOR_9_TLR9_CASCADE	107	1.34	0.001	0.038
WP_GENES_RELATED_TO_PRIMARY_CILIUM_DEVELOPMENT_BASED_ON_CRISPR	91	1.34	0.000	0.038
REACTOME_CELLULAR_RESPONSES_TO_MECHANICAL_STIMULI	96	1.34	0.000	0.038
REACTOME_REGULATION_OF_PD_L1_CD274_POST_TRANSLATIONAL_MODIFICATION	83	1.34	0.000	0.040
PID_MYC_ACTIV_PATHWAY	78	1.34	0.000	0.042
REACTOME_INTEGRATION_OF_ENERGY_METABOLISM	91	1.34	0.000	0.043
REACTOME_SIGNALING_BY_TGFB_FAMILY_MEMBERS	144	1.34	0.012	0.043
REACTOME_ESTROGEN_DEPENDENT_GENE_EXPRESSION	87	1.34	0.000	0.042
KEGG_T_CELL_RECEPTOR_SIGNALING_PATHWAY	91	1.34	0.000	0.042
WP_CLEAR_CELL_RENAL_CELL_CARCINOMA_PATHWAYS	82	1.33	0.000	0.047
REACTOME_CELLULAR_RESPONSE_TO_HEAT_STRESS	95	1.33	0.000	0.046
WP_TNFALPHA_SIGNALING	91	1.33	0.000	0.047
PID_CXCR4_PATHWAY	94	1.33	0.004	0.048
REACTOME_INTERLEUKIN_1_SIGNALING	100	1.33	0.001	0.048
WP_PANCREATIC_ADENOCARCINOMA_PATHWAY	87	1.33	0.000	0.054
REACTOME_MYD88_INDEPENDENT_TLR4_CASCADE	108	1.33	0.000	0.054
REACTOME_MITOCHONDRIAL_TRANSLATION_ELONGATION	87	1.33	0.000	0.053
WP_COMPLEMENT_SYSTEM_IN_NEURONAL_DEVELOPMENT_AND_PLASTICITY	93	1.33	0.004	0.054
WP_P53_TRANSCRIPTIONAL_GENE_NETWORK	83	1.32	0.000	0.058
WP_2Q37_COPY_NUMBER_VARIATION_SYNDROME	104	1.32	0.005	0.058
REACTOME_MITOTIC_SPINDLE_CHECKPOINT	110	1.32	0.005	0.057
REACTOME_CLEC7A_DECTIN_1_SIGNALING	87	1.32	0.003	0.058
WP_HALLMARK_OF_CANCER_SUSTAINING_PROLIFERATIVE_SIGNALING	96	1.32	0.000	0.060
WP_NUCLEAR_RECEPTORS_METAPATHWAY	244	1.32	0.010	0.063
REACTOME_DOWNSTREAM_TCR_SIGNALING	77	1.32	0.000	0.062
REACTOME_ADIPOGENESIS	101	1.32	0.001	0.063
WP_5Q35_COPY_NUMBER_VARIATION	95	1.32	0.003	0.064
REACTOME_TRANSCRIPTIONAL_REGULATION_BY_RUNX3	83	1.32	0.000	0.065
REACTOME_METABOLISM_OF_WATER_SOLUBLE_VITAMINS_AND_COFACTORS	111	1.32	0.012	0.065
KEGG_LEUKOCYTE_TRANSENDOTHELIAL_MIGRATION	102	1.32	0.019	0.065

WP_OREXIN_RECEPTOR_PATHWAY	157	1.32	0.032	0.065
REACTOME_EPH_EPHRIN_SIGNALING	87	1.32	0.001	0.067
REACTOME_REGULATION_OF_HSF1_MEDIATED_HEAT_SHOCK_RESPONSE	76	1.32	0.003	0.067
REACTOME_TP53_REGULATES_METABOLIC_GENES	74	1.31	0.000	0.067
WP_PRADERWILLI_AND_ANGELMAN_SYNDROME	79	1.31	0.000	0.076
REACTOME_ANTIGEN_PROCESSING_CROSS_PRESENTATION	95	1.31	0.009	0.075
REACTOME_P75_NTR_RECEPTOR_MEDIATED_SIGNALLING	95	1.31	0.005	0.077
WP_SPINAL_CORD_INJURY	96	1.31	0.007	0.078
REACTOME_PLATELET_ACTIVATION_SIGNALING_AND_AGGREGATION	235	1.31	0.025	0.079
REACTOME_MITF_M_REGULATED_MELANOCYTE_DEVELOPMENT	135	1.31	0.024	0.082
WP_APOPTOSIS_MODULATION_AND_SIGNALING	85	1.31	0.000	0.082
WP_NONSMALL_CELL_LUNG_CANCER	69	1.31	0.000	0.084
KEGG_PANCREATIC_CANCER	69	1.30	0.000	0.087
REACTOME_LATE_SARS_COV_2_INFECTION_EVENTS	69	1.30	0.000	0.087
REACTOME_TNFR2_NON_CANONICAL_NF_KB_PATHWAY	81	1.30	0.001	0.092
WP_GASTRIN_SIGNALING	104	1.30	0.021	0.096
REACTOME_REGULATION_OF_HOMOTYPIC_CELL_CELL_ADHESION	152	1.30	0.047	0.098
**Downregulated Hallmark Pathways**				
HALLMARK_BILE_ACID_METABOLISM	87	-1.50	0.011	0.048
HALLMARK_FATTY_ACID_METABOLISM	139	-1.44	0.011	0.052
HALLMARK_UNFOLDED_PROTEIN_RESPONSE	108	-1.35	0.056	0.111
**Downregulated C2-CP Pathways**				
REACTOME_ORGANELLE_BIOGENESIS_AND_MAINTENANCE	264	-1.76	0.000	0.000
REACTOME_PROCESSING_OF_CAPPED_INTRON_CONTAINING_PRE_MRNA	285	-1.72	0.000	0.000
WP_INSULIN_SIGNALING	150	-1.71	0.000	0.000
REACTOME_CILIUM_ASSEMBLY	179	-1.71	0.000	0.000
REACTOME_ASPARAGINE_N_LINKED_GLYCOSYLATION	287	-1.69	0.000	0.000
KEGG_UBIQUITIN_MEDIATED_PROTEOLYSIS	129	-1.67	0.000	0.000
REACTOME_MRNA_SPLICING	208	-1.61	0.000	0.004
REACTOME_TRANSPORT_TO_THE_GOLGI_AND_SUBSEQUENT_MODIFICATION	171	-1.61	0.000	0.004
KEGG_PYRIMIDINE_METABOLISM	96	-1.58	0.000	0.008
REACTOME_TRNA_PROCESSING	107	-1.58	0.000	0.008
WP_PYRIMIDINE_METABOLISM	81	-1.57	0.000	0.012
REACTOME_ER_TO_GOLGI_ANTEROGRADE_TRANSPORT	144	-1.56	0.000	0.012
REACTOME_UNFOLDED_PROTEIN_RESPONSE_UPR	91	-1.56	0.000	0.012
REACTOME_TRANSCRIPTIONAL_REGULATION_BY_RUNX2	96	-1.56	0.000	0.013
WP_MIRNA_REGULATION_OF_DNA_DAMAGE_RESPONSE	81	-1.55	0.000	0.016
REACTOME_RECRUITMENT_OF_MITOTIC_CENTROSOME_PROTEINS_AND_COMPLEXES	78	-1.55	0.000	0.016
REACTOME_REGULATION_OF_PLK1_ACTIVITY_AT_G2_M_TRANSITION	84	-1.54	0.000	0.018
REACTOME_ANCHORING_OF_THE_BASAL_BODY_TO_THE_PLASMA_MEMBRANE	93	-1.54	0.000	0.018
REACTOME_AURKA_ACTIVATION_BY_TPX2	69	-1.52	0.000	0.032
PID_SMAD2_3NUCLEAR_PATHWAY	76	-1.51	0.000	0.037
REACTOME_POST_TRANSLATIONAL_MODIFICATION_SYNTHESIS_OF_GPI_ANCHORED_PROTEINS	65	-1.50	0.004	0.044
REACTOME_COPII_MEDIATED_VESICLE_TRANSPORT	65	-1.49	0.000	0.045
PID_P73PATHWAY	73	-1.48	0.008	0.059
BIOCARTA_MAPK_PATHWAY	81	-1.46	0.008	0.082
REACTOME_CARGO_TRAFFICKING_TO_THE_PERICILIARY_MEMBRANE	63	-1.46	0.004	0.084
REACTOME_SUMOYLATION_OF_DNA_DAMAGE_RESPONSE_AND_REPAIR_PROTEINS	73	-1.45	0.009	0.096

nGenes = number of genes, NES = normalized enrichment score, NOM = nominal, FDR = false discovery rate.

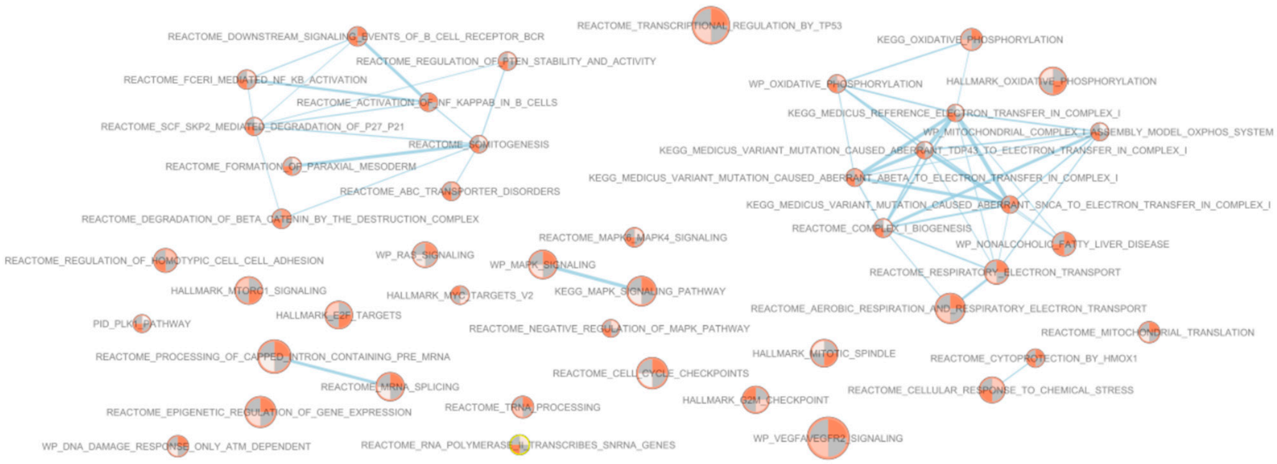

Supplementary Figure S13. Network of significantly enriched pathways or gene sets (FDR <1%) of all significantly upregulated or downregulated genes of all 4 models combined.

Supplementary Methods

RNA isolation and cDNA synthesis

Trizol was added to cell pellets from freshly harvested cell line cultures. RNA was extracted using the miRNeasy Mini or Micro Kit (QIAGEN Benelux B.V. - Belgium, Antwerp, Belgium) in combination with on-column DNase I digestion (RNAse-Free DNase set, Qiagen) according to the manufacturer's guidelines. RNA concentrations were measured by Nanodrop (ThermoFisher Scientific BVBA, Merelbeke, Belgium) and RNA purity and integrity cutoff values were set at a 260/230 ratio and 280/230 ratio of 2. Next, cDNA was synthesized on a Veriti thermal cycler (ThermoFisher Scientific) using 400 ng of RNA and the 5x PrimeScriptTM RT Master Mix (Takara Bio Europe S.A.S.) in a final volume of 10µL. Finally, cDNA was diluted 8-fold to a concentration of 2.5 ng/µL.

qPCR analysis

qPCR reactions were carried out in 96-well plates using 0.3 µM primers, 2x Takyon Low ROX SYBR 2X MasterMix (Eurogentec), 2.5 ng cDNA, and H2O (Sigma-Aldrich) in a 10 µL reaction. Samples were run in duplicate. Briefly, after a heat-activation step (3 min 95 °C), a 2-step real-time protocol of 45 cycles (95 °C 15 sec, 60 °C 60 sec) on a Viia7 analyzer (ThermoFisher) was combined with melting curve analysis (65 °C to 95 °C, gradually increasing with 0.1 °C/sec). Ct thresholds were automatically determined by the QuantStudio™ Real-Time PCR Software.

GAPDH, HPRT-1, and *TBP* were used as housekeeping genes. Primer sequences were in-house developed using the NCBI primer pick tool. All primer pairs were purchased at IDT Technologies. Primer sequences were CTTCTCTCATTCCCCTATGGACA (*CD68*, forward),

GAAGGACACATTGTACTCCACC (*CD68*, reverse), TGCACCACCAACTGCTTAGC (*GAPDH*, forward), GGCATGGACTGTGGTCATGAG (*GAPDH*, reverse), TGACACTGGCAAAACAATGCA (*HPRT-1*, forward), GGTCCTTTTCACCAGCAAGCT (*HPRT-1*, reverse), CACGAACCACGGCACTGATT (*TBP*, forward), and TTTTCTTGCTGCCAGTCTGGAC (*TBP*, reverse). Normalized relative quantity values were calculated according to the delta CT method [22], generating calibrated normalized relative quantity (CNRQ) values.

Protein extraction

Cells were freshly harvested from cell culture and protein lysates were extracted using a Triton X-100 (Sigma-Aldrich). Briefly, wells were incubated with a lysis buffer containing Triton X-100 (1:100) and a protease and phosphatase inhibitor (1:100, Life Technologies Europe BV) for 10 minutes on ice and centrifuges at 4° for 10 minutes at 14000 rpm. Concentration of protein lysate was measured using Pierce™ BCA protein assay quantification kit (Thermo Fisher, Inc.) on a SpectraMax instrument.

Western blot analysis

Western blot analysis was performed according to standard guidelines. Briefly, samples were loaded on a 10% gel and gel-electrophoresis was performed in a Tris-Glycine-SDS (TGS) buffer in a Mini-PROTEAN Tetra System by BioRad. Fluorescent Cy5 was added for the purpose of total protein normalization. Blotting was done on a PVDF membrane using Trans-Blot Turbo Transfer System by BioRad. Primary antibody (mouse recombinant monoclonal CD68 antibody KP1, ab955) and secondary antibody (anti-mouse IgG, HRP-linked, 7076S) were purchased by Abcam and Cell Signaling Technology, respectively, and used in a 1/1000 and 1/3000 dilution in blocking buffer (non-fatty acid milk and Tris-buffered saline with 0.1% Tween® 20 detergent (TBST)). Incubation with the primary antibody was done overnight at 4°C,

followed by 3 washing steps with TBST, and finally incubation with the secondary antibody 1h at room temperature. Imaging was done on a Amersham Imager 680. Normalization was done using ImageQuant analysis software.

Flow cytometry analysis

Staining of surface markers was performed in DPBS with 2% FCS using the antibody-to-cell ratio recommended by the supplier. Identification of different cell populations in the pediatric control samples was done using CD19 (for B-cells), CD3, CD4, CD8, (for T-cells), and CD56 (for NK-cells) staining (Supplementary Methods Table S6). Granulocytes and monocytes were distinguished based on CD45 staining and side scatter-area (SSC-A) (Supplementary Methods Figure S14a). Viable and single cells were selected based on side scatter (SSC) vs forward scatter (FSC) and FSC-A (area) vs FSC-H (height), respectively. Using the Fix&Perm kit (BD Biosciences), cells were permeabilized to evaluate intracellular staining of CD68. Samples were run on a FACSLyric (BD Biosciences) flow cytometry device and analyzed using FACS DIVA software (BD Biosciences) and FlowJo software (TreeStar Inc.).

Intracellular staining of CD68 was evaluated in leukemic bone marrow fractions of patients with pedAML, with identification based on markers of the leukemia-associated immunophenotype (LAIP). The mix of surface LAIP markers included CD45, CD33, CD117, HLA-DR, CD34, and CD38, and was added to all the samples (Supplementary Methods Table S6). To identify the leukemic stem cells, the CD34+ blasts were gated on the CD34+CD38- population. Umbilical cord blood samples were also analyzed on flow cytometry and served as a control for the normal blast population. First, a Lymphoprep (STEMCELL Technologies Lymphoprep, Fisher Scientific) was performed to extract the mononuclear cells of the cord blood samples. CD45 staining

vs SSC-A was used to distinguish the different cell types of the CB samples: embryonic-like stem cells (ELSc), blasts, granulocytes, monocytes, and lymphocytes (Supplementary Methods Figure S14b). CD34 and CD38 antibodies were added to distinguish the CD34+CD38- fraction of the cord blood samples (Supplementary Methods Figure S14c). Cells were permeabilized to evaluate the intracellular staining of CD68 in both leukemic and CB fractions.

All samples were either incubated with a primary anti-CD68 rabbit monoclonal PE-conjugated antibody or with a matched isotype control in the same concentration. CD68 expression was evaluated in the different gated populations. Fold change (FC) differences of median fluorescence intensity (MFI) and percentages of CD68-positive cells were calculated based on comparison with the isotype control.

Supplementary Methods Table S6. Overview of the used antibodies for flow cytometry.

Name of product	Target	Fluorochrome	Company	Catalog #
CD3	CD3	Pacific Blue	BD	558117
CD4	CD4	PE-Cy7	BD	557852
CD8	CD8	APC-H7	BD	641400
CD14	CD14	BV786	BD	563698
CD16	CD16	PE	BD	555407
CD19	CD19	FITC	BD	555412
CD34	CD34	PerCP-Cy5.5	BD	347222
CD38	CD38	APC-H7	BD	656646
CD45	CD45	Pacific Orange	BD	MHCD4530
CD68*	CD68	PE	Life Technologies Europe BV	12-0689-42
Mouse IgG2b kappa isotype control* (eBMG2b)	IgGk2	PE	Life Technologies Europe BV	12-4732-81

*After permeabilization.

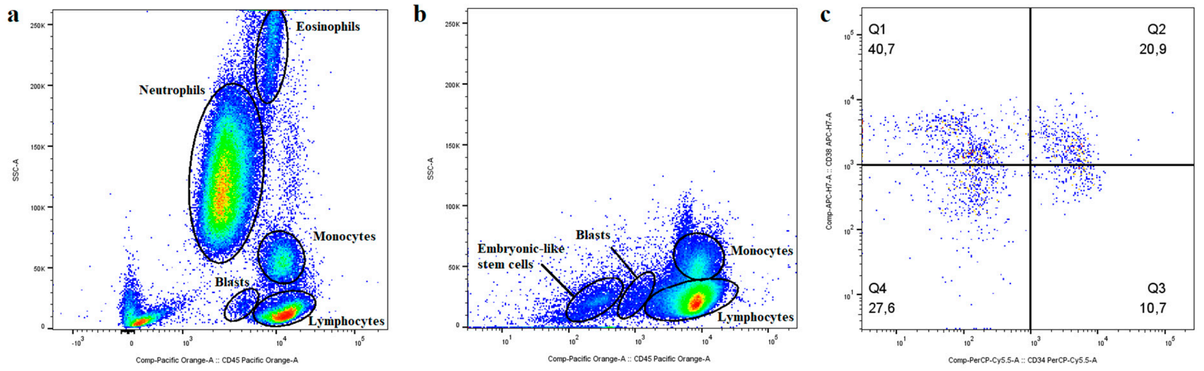

Supplementary Methods Figure S14. a) CD45 staining vs side scatter-area (SSC-A) plot of a pediatric non-hematological non-infectious control sample, with gating of the different blood cell fractions. b) CD45 staining vs SSC-A plot of a cord blood sample with gating of the different blood cell fractions. c) CD34 vs CD38 staining of the blast fraction of a cord blood sample with the CD34+CD38- fraction in the right bottom quadrant (Q3).

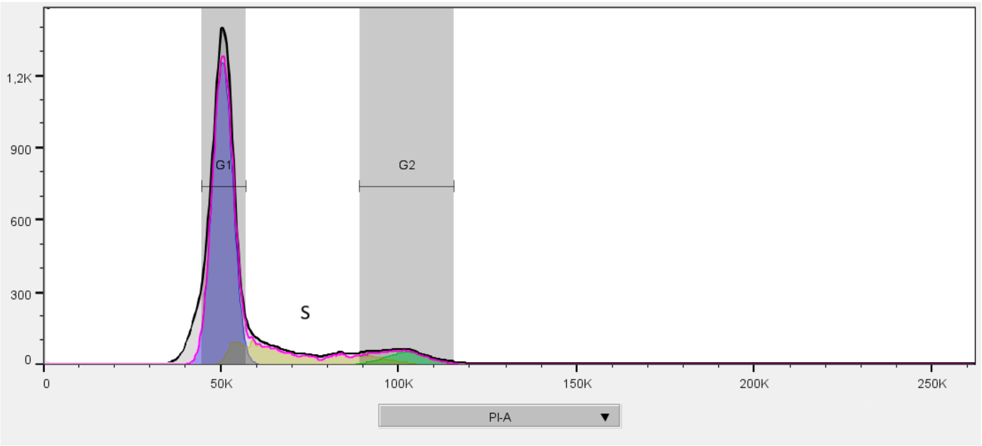

Supplementary Methods Figure S15. Gating strategy in FlowJo of the cell cycle assay using PI (or DAPI) to identify cell cycle phases (G1, S, and G2).

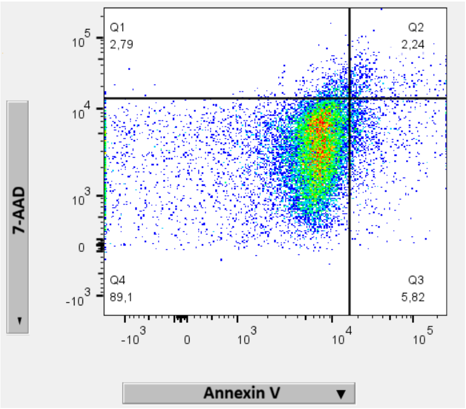

Supplementary Methods Figure S16. Gating strategy on flow cytometry for the apoptosis assay using 7-AAD and Annexin V BV421 to identify live (7-AAD negative, Annexin V negative, Q4), early apoptotic (7-AAD negative, Annexin V positive, Q3), late apoptotic (7-AAD positive, Annexin V positive, Q2), and dead/necrotic cells (7-AAD positive, Annexin V negative, Q1).
